# Altering MRI rotating frame relaxations by changing the truncation level of Hyperbolic Secant pulse

**DOI:** 10.1016/j.jmr.2026.108115

**Published:** 2026-06-17

**Authors:** Sara Ponticorvo, Lin Wu, Andrej Lasica, Hanne Laakso, Dennis J. Sorce, Douglas L. Rothman, Silvia Mangia, Shalom Michaeli

**Affiliations:** a Department of Radiology, Center for Magnetic Resonance Research (CMRR), University of Minnesota, Minneapolis, MN, USA; b Department of Neurology, Charles University, First Faculty of Medicine and General University Hospital, Prague, Czech Republic; c A. I. Virtanen Institute for Molecular Sciences, University of Eastern Finland, Kuopio, Finland; d Independent Researcher, 6 Stonegate Court, Cockeysville, MD 21030, USA; e Department of Radiology and Biomedical Imaging, Magnetic Resonance Research Center (MRRC), Yale University, New Haven, CT, USA

**Keywords:** Truncation level, Adiabaticity, Dipolar relaxations, Exchange, Rotating frames, T_1ρ_, T_2ρ_, Fictitious fields

## Abstract

In this work, we introduce a strategy entitled RETRO (RElaxation dependent on TRuncatiOn) to alter rotating frame relaxations in MRI by changing the truncation level of the amplitude and frequency modulation functions (AM and FM, respectively) of the Hyperbolic Secant (HS) radiofrequency pulses used for achieving adiabatic full inversion. For small truncation levels, as in typical HS pulses, B_1_ is negligible at the beginning of the pulse. In this case, the relaxation process during the pulse is characterized solely by the longitudinal relaxation time constant ${\text{T}}_{1\rho ,1}(\text{t})$ when magnetization M is initially aligned with the effective magnetic field $B_{\text{eff}}^{\left(1\right)}(\text{t})$, or by the transverse relaxation time constant ${\text{T}}_{2\rho ,1}(\text{t})$ when M is initially placed on a plane perpendicular to $B_{\text{eff}}^{\left(1\right)}(\text{t})$. However, when the truncation level is non-zero, an instantaneous non-zero B_1_ field is formed at the beginning of the HS pulse, leading to relaxation that comprises both ${\text{T}}_{1\text{p},1}(\text{t})$ and ${\text{T}}_{2\rho ,1}(\text{t})$ pathways, with relative contributions depending on the truncation level. Here, after introducing the basis of the RETRO strategy, we provide theoretical descriptions of homonuclear dipolar relaxations between like ½ spins in a weak collision regime, along with exchange-induced relaxations between two magnetic spin populations with different chemical shifts (Δω≠0) during the pulses. In addition, we describe the features of *in vivo* RETRO contrasts obtained in the rodent brain with different pulse durations and truncation levels, and demonstrate that high truncation levels allow shortening pulse duration while maintaining RF amplitudes suitable for *in vivo* investigations. We conclude that, by enabling a wide range of HS pulse configurations to achieve full adiabatic inversion, the RETRO strategy generates flexible and robust MRI contrasts that can probe fast relaxing components typically not accessible *in vivo* with conventional adiabatic ${\text{T}}_{1,2\rho }$ techniques.

## 1. Introduction

Studies of slow and ultraslow motions in solids have been pursued since the early days of NMR [1–9]. High field NMR approaches that characterize slow motions of the spin dynamics generally evaluate NMR relaxations in multifold rotating frames [10], and they may employ separate coils and amplifiers for detecting relaxations in high-rank rotating frames [3,4,11–13], or audio frequency for exciting the spin ensemble in solids [14]. While most solid-state NMR methods had been developed for applications in material science and structural biology, recently there has been increased interest to develop safe and non-invasive rotating frame relaxation methods applicable to *in vivo* MRI [15–18].

Inspired by previous solid-state NMR works, we introduced the RAFFn technique (Relaxations Along a Fictitious Field in the rotating frame of rank n) that also operates in multifold rotating frames [19–21], and is predominantly sensitive to molecular motional regimes in the ~0.5−2ms time scale in applications *in vivo*, for instance allowing assessment of myelin content and integrity [22,23], or myocardial infarct [24] and calcifications [25]. The RAFFn technique complements the repertoire of adiabatic ${\text{T}}_{1\rho }$ and ${\text{T}}_{2\rho }$ methods that have shown great potential as tissue markers of pathology [26–31], as well as for protein dynamics characterization, allowing to probe “hidden dynamics” of the molecular functional groups in the presence of singularities in MR dispersion profiles [32–36]. Adiabatic ${\text{T}}_{1\rho }$ and ${\text{T}}_{2\rho }$ are particularly appealing for applications *in vivo* because they can be tuned to a broad range of motional regimes at high magnetic fields (3 T and above), and are highly tolerant to B_0_ and B_1_ variations. Yet, for human studies, the safety limits on specific absorption rates (SAR), along with limited maximum RF amplitudes achievable on clinical scanners, restrict the viable parameter settings for achieving adiabaticity to pulse durations in the range of several *ms* and to low values of time-bandwidth product (R-value), thus hampering the access to fast-relaxing spin packets in sub-millisecond timescales. The non-adiabatic RAFFn methods [19–21] were developed to enhance sensitivity to slow motional regimes while reducing RF peak requirements. However, they are characterized by narrower bandwidth (BW) as compared to adiabatic ${\text{T}}_{1\rho }$ and ${\text{T}}_{2\rho }$ and require precise RF power calibrations, which ultimately warrants dedicated B_1_ shimming procedures especially at ultra-high magnetic fields. Therefore, further developments of rotating frame methods that enable shorter pulse durations ideally in a sub-milliseconds time scale at sufficiently low power, broad BW, and tolerance to B_0_ and B_1_ variations are desirable, motivating the current study.

With the goal of expanding the arsenal of MR methods for probing fast relaxing spins *in vivo*, here we introduce the rotating frame approach entitled RETRO (RElaxation dependent on TRuncatiOn), which is based on the use of truncated versions of radiofrequency (RF) pulses of the Hyperbolic Secant (HS) family. Using consecutive transformations to superadiabatic frames, we demonstrate that, with accurate power calibrations, the truncated HS pulses provide a complete inversion of the magnetization while satisfying adiabatic conditions, despite M never being locked along the $B_{\text{eff}}^{\left(1\right)}(\text{t})$ during the inversion. To describe relaxation during the truncated HS pulses, we first evaluated homonuclear dipole-dipole relaxations between like spins ½ during the pulses in weak collision regime (WCR), for which we utilized Wigner transformations between frames and employed trigonometric relationships [37]. In addition, we evaluated relaxations induced by anisochronous exchange between spins with different chemical shifts. Then, we further characterized the *in vivo* relaxation contrasts in the rodent brain during truncated HS1 pulses with different pulse durations ranging from sub-milliseconds to several milliseconds, and different readouts. Finally, correlation analyses between relaxation time constants during truncated HS1 pulses and the myelin content in the rat brain presented in our prior work [22] provided preliminary evidence that RETRO could be sensitized to the slow motional spins associated with myelin. We also speculate that RETRO may provide a mean to highlight the distributions of other tissue component beyond myelin, possibly leading to novel insights into tissue structure and function.

## 2. Relaxation during frequency swept pulses

**Fig. 1.**
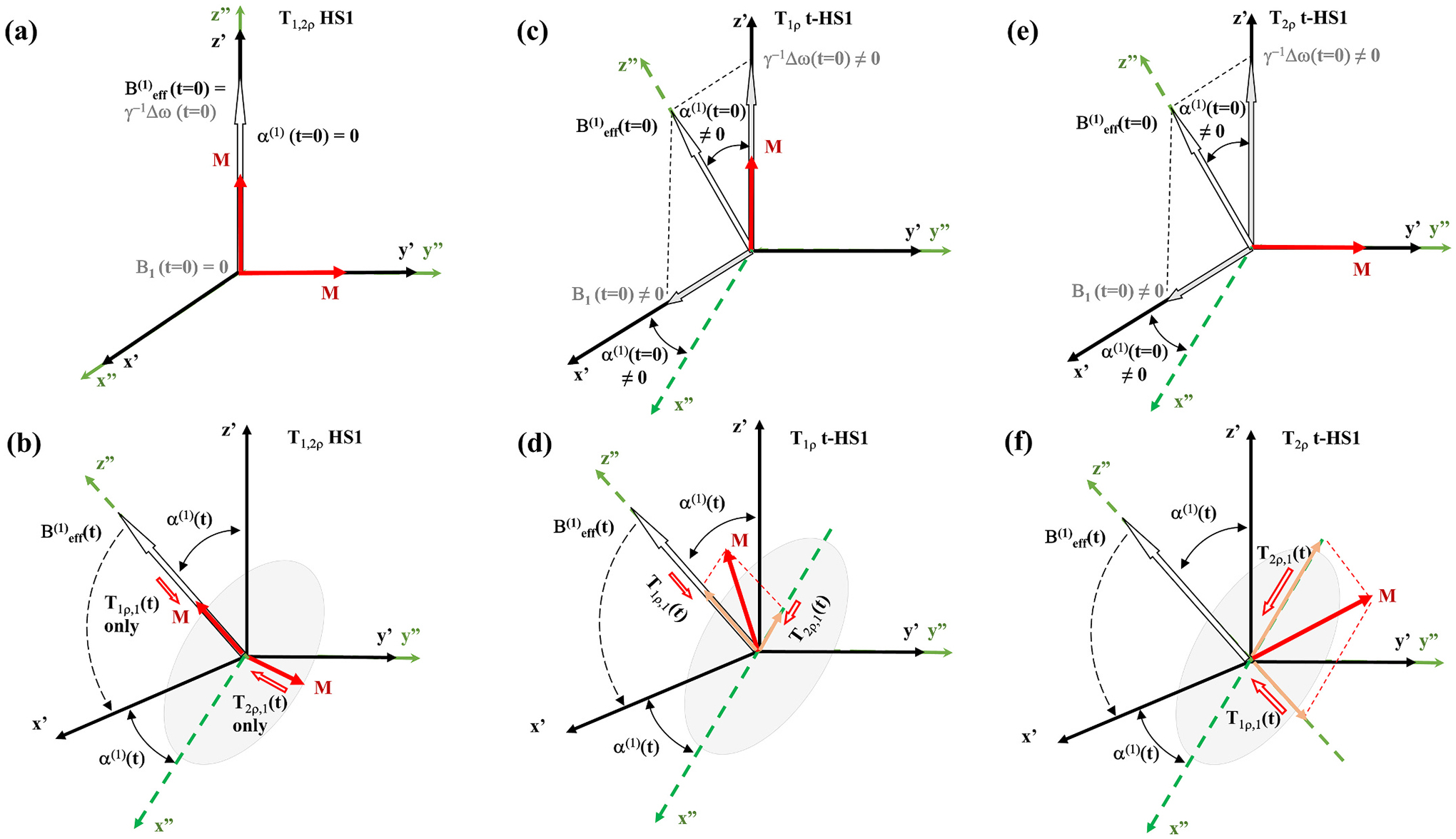
Vector diagram of the relaxations during HS1 and t-HS1 pulses. (a) Magnetization M is initially unperturbed and oriented along z′ axis of the first rotating frame (FRF), or placed in the x′y′ plane following excitation by a 90° pulse. (b) With HS1 pulse, at t = 0, the RF amplitude is zero while the offset frequency has a non-zero value. Therefore, $B_{\text{eff}}^{\left(1\right)}$ is oriented along the z′ axis at the beginning of the HS1 pulse. During the HS1 pulse operating in the adiabatic regime, if M is initially collinear with $B_{\text{eff}}^{\left(1\right)}(\text{t})$, it will follow $B_{\text{eff}}^{\left(1\right)}(\text{t})$. Thus, the relaxation process is characterized by the time constant ${\text{T}}_{1\rho ,1}(\text{t})$. On the other hand, if M is initially placed in the x′y′ plane, it undergoes precession on a plane perpendicular to $B_{\text{eff}}^{\left(1\right)}(\text{t})$, and relaxation is solely $T_{2\rho ,1}(\text{t})$. (c,d) With t-HS1, due to non-zero magnitude of $B_{1}$ at t = 0 and a non-zero value of the $\gamma^{-1}\Delta \omega ,B_{\text{eff}}^{\left(1\right)}$ is tilted to the angle $\alpha^{\left(1\right)}$ at t = 0, which is the angle between M and $B_{\text{eff}}^{\left(1\right)}$ at the beginning of the t-HS1 pulse. Note that M is never collinear with $B_{\text{eff}}^{\left(1\right)}(\text{t})$, and the relaxation process is comprised of both ${\text{T}}_{1\rho ,1}$ and ${\text{T}}_{2\rho ,1}$ relaxations. (e,f) When M is originally located in the x′y′ plane following the excitation by 90° pulse, M follows $B_{\text{eff}}^{\left(1\right)}(\text{t})$ and undergoes precession on a plane which makes an angle $90{}^{\circ}+\alpha^{\left(1\right)}(\text{t})$ with the z″ axis. The relaxation process is predominantly ${\text{T}}_{2\rho ,1}$ with some contribution from ${\text{T}}_{1\rho ,1}$ because of non-zero angle $\alpha^{\left(1\right)}(\text{t})$.

Rotating frame relaxation methods based on RF pulses with both amplitude and frequency modulations (AM and FM) are classified as adiabatic and non-adiabatic techniques. The former methods, adiabatic ${\text{T}}_{1\rho }$ and ${\text{T}}_{2\rho }$, utilize frequency swept (FS) RF pulses that achieve adiabatic full passage (AFP) [17,37–41]. The latter methods, entitled RAFFn, utilize RF pulses that operate under non-adiabatic conditions, inducing relaxations in multi-fold rotating frames due to the generation of fictitious magnetic fields [19–22]. The description of spin dynamics in higher order rotating frames is not new in NMR; originally, spin dynamics in doubly rotating frame during frequency swept RF pulses was described in the pioneering works by Bendall et al., where the formation of fictitious magnetic fields was evaluated during the amplitude and frequency modulated pulses [42,43]. During AFP pulses, the relaxation of the spin ensemble is determined by the relative orientations of magnetization M and the effective magnetic field $B_{\text{eff}}^{\left(1\right)}(\text{t})$ of the first rotating frame (FRF). When the adiabatic condition $\left|\gamma^{-1}\text{d}\alpha^{\left(1\right)}(\text{t})/\text{dt}\right|\ll {\text{B}}_{\text{eff}}^{\left(1\right)}(\text{t})$ is well satisfied, the magnetization M, if initially collinear with or in a plane perpendicular to $B_{\text{eff}}^{\left(1\right)}(\text{t})$ (Fig. 1a), will remain approximately aligned with $B_{\text{eff}}^{\left(1\right)}(\text{t})$ (locked) or in a plane perpendicular to $B_{\text{eff}}^{\left(1\right)}(\text{t})$ during the application of AFP pulse, respectively (Fig. 1b). Here, $\alpha^{\left(1\right)}(\text{t})$ is the angle between the $B_{\text{eff}}^{\left(1\right)}(\text{t})$ and the axis of quantization of the FRF z′, $\gamma^{-1}\text{d}\alpha^{\left(1\right)}(\text{t})/\text{dt}$ is the fictitious magnetic field, and the $\text{d}\alpha^{\left(1\right)}(\text{t})/\text{dt}$ is the angular velocity of the effective field. In the former case, the relaxation process is characterized by the time constant ${\text{T}}_{1\rho }(\text{t})$ in the FRF, here noted as ${\text{T}}_{1\rho ,1}(\text{t})$ [40]. In the latter case, M undergoes Rabi precession in a plane perpendicular to $B_{\text{eff}}^{\left(1\right)}(\text{t})$ during the adiabatic passage (Fig. 1b), and thus relaxation is characterized by the time constant ${\text{T}}_{2\rho }(\text{t})$ in the FRF, here noted as ${\text{T}}_{2\rho ,1}(\text{t})$ [17,38,39].

Recently, we have shown that for the general treatment of relaxation during HS pulses of the HSn family (where n is stretching factor), the fictitious field generated due to the time evolution of $B_{\text{eff}}^{\left(1\right)}(\text{t})$ needs to be taken into account, and thus treatment in the multi-fold rotating frames is necessary to adequately describe relaxation [17,37]. The contribution of the fictitious field to the relaxation functions had been shown to depend on the modulation functions of the pulses and on pulse parameter’s settings. In the case of HS pulse, here considered with stretching factor n=1 (HS1), with low truncation level of the AM at the boundaries of the pulses, this contribution was found to be insignificant [37]. On the other hand, when the truncation level becomes larger, a non-zero value of $B_{1}$ is formed at t=0 of the pulse, and the $B_{\text{eff}}^{\left(1\right)}(\text{t})$ forms a non-zero angle $\alpha^{\left(1\right)}$ with the z′ axis at the beginning of the pulse (Fig. 1c). Magnetization M is thus never collinear with $B_{\text{eff}}^{\left(1\right)}(\text{t})$ during the pulse (Fig. 1d), however it can be decomposed in two components which respectively undergo ${\text{T}}_{1\rho ,1}$ and ${\text{T}}_{2\rho ,1}$ relaxations (Fig. 1d). When M is originally located in the x′y′ plane following the excitation by 90° pulse, M follows $B_{\text{eff}}^{\left(1\right)}(\text{t})$ and undergoes precession on a plane which makes an angle $90{}^{\circ}+\alpha^{\left(1\right)}(\text{t})$ with the z″ axis of quantization (Fig. 1e), and the relaxation process is again comprised of ${\text{T}}_{2\rho ,1}$ and ${\text{T}}_{1\rho ,1}$ relaxations (Fig. 1f).

In the RETRO methodology, different MRI relaxation contrasts are generated by changing the truncation level of the AM and FM functions of AFP pulses. Accordingly, we will refer to truncated HS1 (t-HS1) pulses when considering HS1 pulses with non-negligible truncation levels. In addition, for consistency with the conventional approaches of adiabatic ${\text{T}}_{1\rho }$ and ${\text{T}}_{2\rho }$ [16], we will refer to ${\text{T}}_{1\rho }$ or ${\text{T}}_{2\rho }$ depending on whether M is initially collinear with z′ or excited to the x′y′ plane, respectively. Importantly, both ${\text{T}}_{1\rho }$ and ${\text{T}}_{2\rho }$ during t-HS1 pulses comprise ${\text{T}}_{1\rho ,1}$ and ${\text{T}}_{2\rho ,1}$ channels, each with different relative contributions.

Since a complete solution of the relaxations during the FS pulses requires treatment in high-order rotating frames [37] due to the presence of a fictitious field component that contributes to the relaxations *via* $\text{J}\left(\omega_{\text{eff}}^{\left(2\right)}\right)$, all calculations were performed here in the second rotating frame (SRF). The relaxation processes during RF pulses depend on pulse parameter’s settings, and the truncation level plays a pivotal role as it defines the relative weightings of the ${\text{T}}_{1\rho ,1}$ versus ${\text{T}}_{2\rho ,1}$ relaxation pathways. In fact, an adiabatic ${\text{T}}_{1\rho }$ configuration can even become solely ${\text{T}}_{2\rho }$-weighted, as it occurs with the family of B_1_-insensitive rotation (BIR) pulses (BIR-1 and BIR-4), obtained with multiple asymmetrically truncated HS1 pulses (for details see [44] and references therein). With BIR pulses, the RF amplitude is maximal at the beginning of the pulse, while the frequency sweep starts from zero. Therefore, $B_{\text{eff}}^{\left(1\right)}(\text{t})$ is instantaneously switched to its maximal amplitude in the x′y′ plane at the beginning of the RF pulse [44]. When the magnetization M is initially not perturbed and oriented along the z′ axis of the FRF, it undergoes precession in a plane perpendicular to the $B_{\text{eff}}^{\left(1\right)}(\text{t})$ during the BIR pulses. Thus, the relaxation process during the pulse is characterized solely by time constant ${\text{T}}_{2\rho ,1}\left(\text{t}\right)$ [39,44,45].

Description of the relaxation during frequency swept pulses requires consideration of weak, intermediate and strong collision regimes (WCR, ICR and SCR respectively), which are defined by the relationships $\tau_{\text{c}}\ll {\text{T}}_{\text{m}}$ for WCR, $\tau_{\text{c}}\sim {\text{T}}_{\text{m}}$ for ICR and $\tau_{\text{c}}\gg {\text{T}}_{\text{m}}$ for SCR [1,2,46]. Here $\tau_{\text{c}}$ represents the correlation time between consecutive jumps or reorientations of the spin Hamiltonian, and T_m_ is the spin-spin relaxation time constant of the lattice environment required for the spin system to achieve equilibrium in the rotating frame, or the time required for thermal mixing of dipolar and Zeeman heat reservoirs [1,2]. For *in vivo* tissue characterization, consideration of WCR is most common [47,48]. The theoretical formalism developed within the WCR limits is indeed useful for assessing the fast motion of molecules or functional groups [49,50]. On the other hand, the ICR and SCR are less characterized for *in vivo* applications, although their assessment could be relevant to tissue contrast and pathology. In addition, relaxations *in vivo* require consideration of multiple pools of water. The most parsimonious model is a two-pool model which involves two different populations of protons, by convention associated with free water and bound water, characterized by slow and fast relaxation rates, respectively, for free precession and rotating frame relaxation channels. Relaxation parameters and pool sizes can be determined by fitting the bi-exponential SI evolution, provided that the number and spacing of the sampling points is adequate, which implies that the duration of the FS pulses needs to be as short as possible in order to sample fast-relaxing spins in rotating frame experiments. Moreover, since fast relaxing spins can be filtered out by the sequence readouts, we recently developed a method for asymptotic ${\text{T}}_{1\rho }$ relaxation measurements using Zero-TE MB-SWIFT (Multi-Band SWeeping Imaging with Fourier Transformation) as a readout [51–53]. We have shown that the short relaxing component can be determined from the slope of the SI decay curve, using the initial data points plotted on the logarithmic scale [54]. To capture fast relaxing spins in bovine tendon specimens, AFP pulses with 0.3 ms pulse duration and with power settings around 10 kHz were used [54]. However, these settings are impractical for *in vivo* applications due to potential tissue damage, which motivated the development of the RETRO methodology.

## 3. The truncated HS1 pulses used for RETRO

The amplitude and frequency modulation functions of the conventional HS1 pulse are defined as follows [44]:

(1) $$\omega_{1}(t)=\omega_{1}^{max}\;\text{sech}\left(\beta \left(2t/T_{p}-1\right)\right)$$

and

(2) $$\omega_{RF}(t)-\omega_{c}=A\text{tanh}\left(\beta \left(2t/T_{p}-1\right)\right).$$

**Fig. 2.**
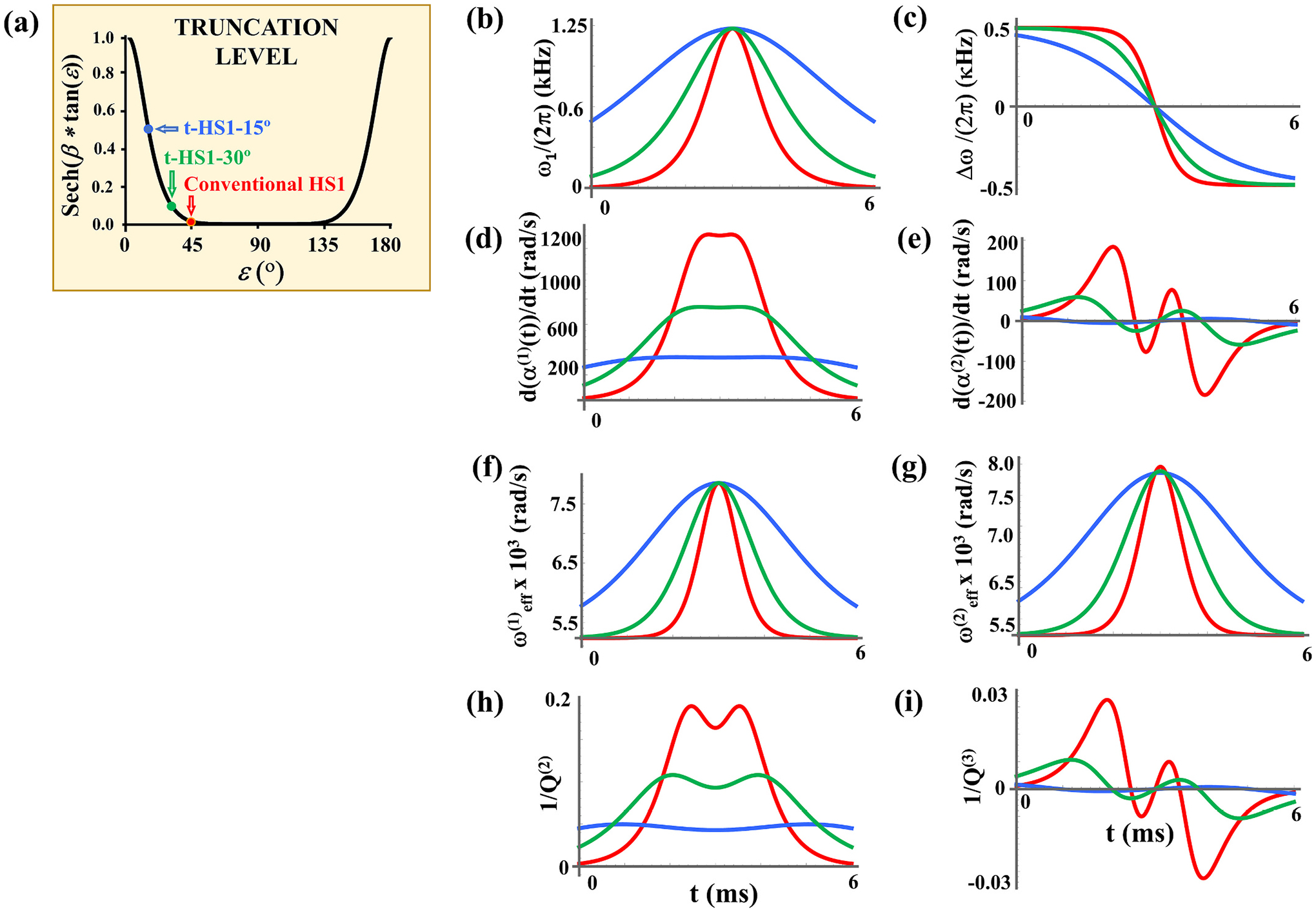
HS pulse at different levels of truncation. (a) Dependence of the truncation level on ε; Amplitude (b) and frequency (c) modulation functions of the HS1 (red line), t-HS1-15° (blue line), and t-HS1-30° (green line) pulses. Pulse parameters are: peak RF amplitude $\omega_{1}^{\text{max}}/(2\pi )=1.25\;\text{kHz},\;\text{sech}(\beta )=0.01$, and ε=45°, 15° and 30° for HS1, t-HS1-15° and t-HS1-30° pulses, respectively. Truncation levels of HS1, t-HS1-15° and t-HS1-30° pulses are 0.01, 0.46 and 0.09, respectively, and time-bandwidth products $\left(\text{R}\equiv {\text{AT}}_{\text{p}}/\pi \right)$ are 10, 9 and 10, respectively; (d,e) time dependence of $\text{d}\alpha^{\left(1\right)}(\text{t})/\text{dt}$ and $\text{d}\alpha^{\left(2\right)}(\text{t})/\text{dt}$ during the HS1, t-HS1-15° and t-HS1-30° pulses; (f,g) time dependence of the effective frequencies in the first and second rotating frames, $\omega_{\text{eff}}^{\left(1\right)}(\text{t})$ and $\omega_{\text{eff}}^{\left(2\right)}(\text{t})$, during the HS1, t-HS1-15° and t-HS1-30 pulses; (h,i) inverse of the adiabaticity and superadiabaticity factors, 1/Q^(2)^ and 1/Q^(3)^, in the second and the third rotating frames during the HS1, t-HS1-15° and t-HS1-30° pulses.

Here, β is the truncation factor, A is the amplitude of the frequency sweep in rad/s with the bandwidth of the pulse being BW = 2A, T_p_ is the pulse duration, $\text{t}\in \left[0,{\text{T}}_{\text{p}}\right],\omega_{\text{c}}$ is the carrier frequency (at the center of the bandwidth of interest), and $\omega_{1}^{\text{max}}$ is the maximum value of $\omega_{1}(\text{t})$. The truncation level, Sech(β), of the AM function of the conventional adiabatic HS1 pulse is generally small at the pulse’s boundaries, namely between 0.001 and 0.01. Such a small truncation level ensures collinearity of $B_{\text{eff}}^{\left(1\right)}(\text{t})$ with M during the adiabatic inversion. On the other hand, in the RETRO strategy we purposely introduced a modulation of the truncation level according to tan(ε) function with 0≤ε≤180° (Fig. 2a), leading to the following AM and FM functions:

(3) $$\omega_{1}\left(t\right)=\omega_{1}^{max}\;\text{sech}\left(\beta \text{tan}(\varepsilon )\left(2t/T_{p}-1\right)\right),$$

and

(4) $$\omega_{RF}\left(t\right)-\omega_{c}=A\text{tanh}\left(\beta \text{tan}\left(\varepsilon \right)\left(2t/T_{p}-1\right)\right).$$

It can be easily realized that with ε=45° one obtains the conventional modulation functions of the HS1 pulse. Notably, the tan(ε) function approaches infinity for any $\varepsilon ={\text{k}}^{*}\pi /2$, where k = 1,3,5… is an odd number. Therefore, the following limitation should be imposed: $\varepsilon \neq {\text{k}}^{*}\pi /2$. It should also be noted that the tan(ε) function used in this work is just one example for varying the level of truncation; other functions (*e.g*., a simple linear function) could be utilized instead.

It should be noted that the modulations of the AM and FM functions according to tan(ε) separately and together result in different evolutions of M during the HS1 pulse, ultimately leading to different relaxation processes. For a general description of relaxation during AM and FM pulses, the sequential transformations from the FRF to a multifold rotating frames of rank σ should be used [37]. The angle between the z′ axis of quantization of the FRF and the $B_{\text{eff}}^{\left(1\right)}(\text{t})$ is defined as follows [44]:

(5) $$\alpha^{\left(\sigma =1\right)}\left(t\right)={\text{tan}}^{-1}\left(\frac{\omega_{1}\left(t\right)}{\Delta \omega \left(t\right)}\right),$$

where $𝚫𝛚(\text{t})=\left(\omega_{0}-\omega_{\text{RF}}(\text{t})\right){\hat{z}}^{\prime }$ is the time-dependent frequency offset in the FRF and $\omega_{1}(\text{t})$ is the time dependent RF pulse amplitude in frequency units. The effective frequency is also time dependent, and is given by:

(6) $$\gamma {B^{\left(1\right)}}_{\text{eff}}\left(t\right)={\omega^{\left(1\right)}}_{\text{eff}}\left(t\right)=\sqrt{{\left(\omega_{1}\left(t\right)\right)}^{2}+(\Delta \omega (t){)}^{2}}.$$

To transform to the SRF(σ=2), we must introduce another angular quantity and a corresponding vectorial contribution to the effective field [55]. To accomplish this, we define:

(7) $$\alpha^{\left(2\right)}(t)={\text{tan}}^{-1}\left(\frac{d\alpha^{\left(1\right)}(t)/dt}{\omega^{\left(2\right)}eff(t)}\right),$$

and

(8) $${\omega^{\left(2\right)}}_{\text{eff}}(t)=\sqrt{{\left({\omega^{\left(1\right)}}_{\text{eff}}(t)\right)}^{2}+{\left(d\alpha^{\left(1\right)}(t)/dt\right)}^{2}}.$$

Further transformations to a multifold rotating frame of rank σ are performed as follows [19,37,55]:

(9) $$\begin{array}{l} \alpha^{\left(3\right)}(t)={\text{tan}}^{-1}\left(\frac{\frac{d\alpha^{\left(2\right)}(t)}{dt}}{{\omega^{\left(3\right)}}_{eff}(t)}\right)\\ \alpha^{\left(4\right)}(t)={\text{tan}}^{-1}\left(\frac{d\alpha^{\left(3\right)}(t)/dt}{{\omega^{\left(4\right)}}_{eff}(t)}\right)\\ \ldots \\ \alpha^{\left(\sigma \right)}(t)={\text{tan}}^{-1}\left(\frac{d\alpha^{\left(\sigma -1\right)}(t)/dt}{{\omega^{\left(\sigma \right)}}_{ef\text{f}}(t)}\right), \end{array}$$

and

(10) $$\begin{array}{l} {\omega^{\left(3\right)}}_{eff}(t)=\sqrt{{\left({\omega^{\left(2\right)}}_{eff}(t)\right)}^{2}+{\left(d\alpha^{\left(2\right)}(t)/dt\right)}^{2}}\\ {\omega^{\left(4\right)}}_{eff}(t)=\sqrt{{\left({\omega^{\left(3\right)}}_{eff}(t)\right)}^{2}+{\left(d\alpha^{\left(3\right)}(t)/dt\right)}^{2}}\\ \ldots \\ {\omega^{\left(\sigma \right)}}_{eff}(t)=\sqrt{{\left({\omega^{\left(\sigma -1\right)}}_{eff}(t)\right)}^{2}+{\left(d\alpha^{\left(\sigma -1\right)}(t)/dt\right)}^{2}}. \end{array}$$

## 4. Adiabaticity and superadiabaticity in multifold rotating frames

Several factors can cause misalignment of the magnetization vector M and the effective field $B_{\text{eff}}^{\left(1\right)}(\text{t})$ during the adiabatic ${\text{T}}_{1\rho }$ measurement. The first scenario occurs when M is aligned with $B_{\text{eff}}^{\left(1\right)}(\text{t})$ at t = 0 (i.e., the level of truncation is small), but fails to follow $B_{\text{eff}}^{\left(1\right)}(\text{t})$ perfectly during the pulse due to an insufficiently satisfied adiabatic condition. In this case, the relaxation process is governed by a combination of ${\text{T}}_{1\rho }$ and ${\text{T}}_{2\rho }$ pathways. A second scenario arises when M is not parallel with $B_{\text{eff}}^{\left(1\right)}(\text{t})$ at t = 0 of the AFP pulse due to a large truncation level (*e.g*., as in the RETRO technique), which creates a non-zero angle between the effective field and z′ axis (Fig. 1b). Here relaxation is also dictated by both ${\text{T}}_{1\rho }$ and ${\text{T}}_{2\rho }$ pathways.

When the adiabaticity is violated, causing M to not be perfectly collinear with $B_{\text{eff}}^{\left(1\right)}(\text{t})$ during inversion, relaxation should be evaluated in the rotating frames of ranks σ≥2 to account for the contributions from both ${\text{T}}_{1\rho ,\sigma }$ and ${\text{T}}_{2\rho ,\sigma }$ channels due to the formation of fictitious magnetic fields [37,56]. Another factor to consider during AFP pulse is the formation of geometric phases when the adiabatic condition is well-satisfied, or sub-geometric phases when it is violated [56]. The concept of the geometric phases was introduced by Berry for the adiabatic evolution of spin Hamiltonian [57,58], and subsequently was generalized by Aharonov and Anandan for the non–adiabatic evolution of a spin ensemble [59]. We have shown that sub-geometric phases during the FS pulses operating in the non-adiabatic regime depend on the initial conditions of the spinor, leading to changes of spin polarization influenced by amplitude modulation (AM) and frequency modulation (FM) [60]. Overall, this suggests that corrections for the sub-geometric phases during the evolution of the density matrix may be required to accurately describing the relaxation [56,60]. Yet, for simplicity, we provide only a general description for evaluating the conditions of inversion, omitting specific relaxation pathway and formation of geometric or sub-geometric phases during the adiabatic or non-adiabatic evolution of spin Hamiltonian, respectively.

By convention, to quantitatively evaluate the adiabatic condition during the AM and FM pulse, the adiabaticity factor Q is defined in the FRF (σ=1) as the ratio of the effective frequency $\omega_{\text{eff}}^{\left(1\right)}(\text{t})$ and the $|\text{d}\alpha^{\left(1\right)}(\text{t})/\text{dt}|$, given by:

(11) $$Q(t)=\frac{{\omega^{\left(1\right)}}_{\text{eff}}(t)}{\left|d\alpha^{\left(1\right)}(t)/dt\right|}.$$

The adiabatic condition is usually considered to be well satisfied for the Q factors greater than 5 [61]. It has been realized, however, that this requirement is not strict and that perfect magnetization inversion during AFP could be obtained with an adiabaticity factor < 5 [61]. It has been shown that the condition of perfect magnetization inversion during an AFP pulse is not primarily dictated by the locking of M along the $B_{\text{eff}}^{\left(1\right)}(t)$ in the FRF, since this is practically never the case during the application of AFP pulses. The conditions of complete inversion of M were recently evaluated by Deschamps et al. [62] based on a seminal contribution by Berry [58], showing that if the system is adiabatic in any of the superadiabatic frames, this allow for perfect inversion of the magnetization M since it is locked to the $B_{\text{eff}}^{\left(𝛔\right)}(t)$ in the superadiabatic frame [62]. Thus, the adiabaticity factor is defined in the superadiabatic rotating frames of rank σ as follows:

(12) $$Q^{\left(\sigma \right)}(t)=\frac{{\omega^{\left(\sigma \right)}}_{eff}(t)}{\left|d\alpha^{\left(\sigma \right)}(t)/dt\right|},$$

with σ greater than 1, where Eqs. (9), (10) are used to obtain $\omega_{\text{eff}}^{\left(\sigma \right)}(\text{t})$ and $\text{d}\alpha^{\left(\sigma \right)}(\text{t})/\text{dt}$. Full inversion is achieved whenever M is perfectly locked in any of superadiabatic frames [62], according to [62]:

(13) $$\frac{1}{Q^{s}}=\underset{\sigma }{min}\left[\underset{t\in [0,\tau ]}{max}\frac{1}{Q^{\left(\sigma \right)}(t)}\right].$$

Here, σ indicates the rank of the superadiabatic frame, and ${\text{Q}}^{\text{s}}$ is the superadiabatic factor. Thus, Eq. (13) describes the condition for the optimal inversion of M, specifically indicating that $1/{\text{Q}}^{\text{s}}$ should be sufficiently small at the end of the pulse to achieve complete inversion of M.

For visualization purposes, Fig. 2a shows the dependence of the truncation level on ε. For calculations the Sech ($\beta^{*}\text{tan}(\varepsilon )$) function with β=5.2983 (*i.e*., Sech (β)=0.01) was used. Fig. 2b,c demonstrates the AM and FM functions of the classical HS1 pulse (*i.e*., with truncation level = 0.01, corresponding to ε=45°) and of the t-HS1-15° and t-HS1-30° pulses (*i.e*., HS1 pulses with ε values = 15° and 30°, leading to truncation levels = 0.46 and 0.09, respectively). The truncation levels of the HS1 pulse lead to different $\alpha^{\left(1\right)}(\text{t})$ and $\alpha^{\left(2\right)}(\text{t})$ functions, ultimately resulting in differences between $\text{d}\alpha^{\left(1\right)}(\text{t})/\text{dt}$ and $\text{d}\alpha^{\left(2\right)}(\text{t})/\text{dt}$ (Fig. 2d,e) and the effective frequencies $\omega_{\text{eff}}^{\left(1\right)}(\text{t})$ and $\omega_{\text{eff}}^{\left(2\right)}(\text{t})$ in the FRF and SRF (Fig. 2f,g), respectively, which lead to the adiabaticity and superadiabaticity factors shown in Fig. 2h,i. The latter plots show that adiabaticity is preserved for t-HS1-15° and 30° pulses despite high truncation levels. Additionally, $1/{\text{Q}}^{\text{s}}$ is sufficiently small in the SRF and TRF at the end of the pulses to satisfy the condition of a complete inversion of M (see Eq. (13)) [62].

**Fig. 3.**
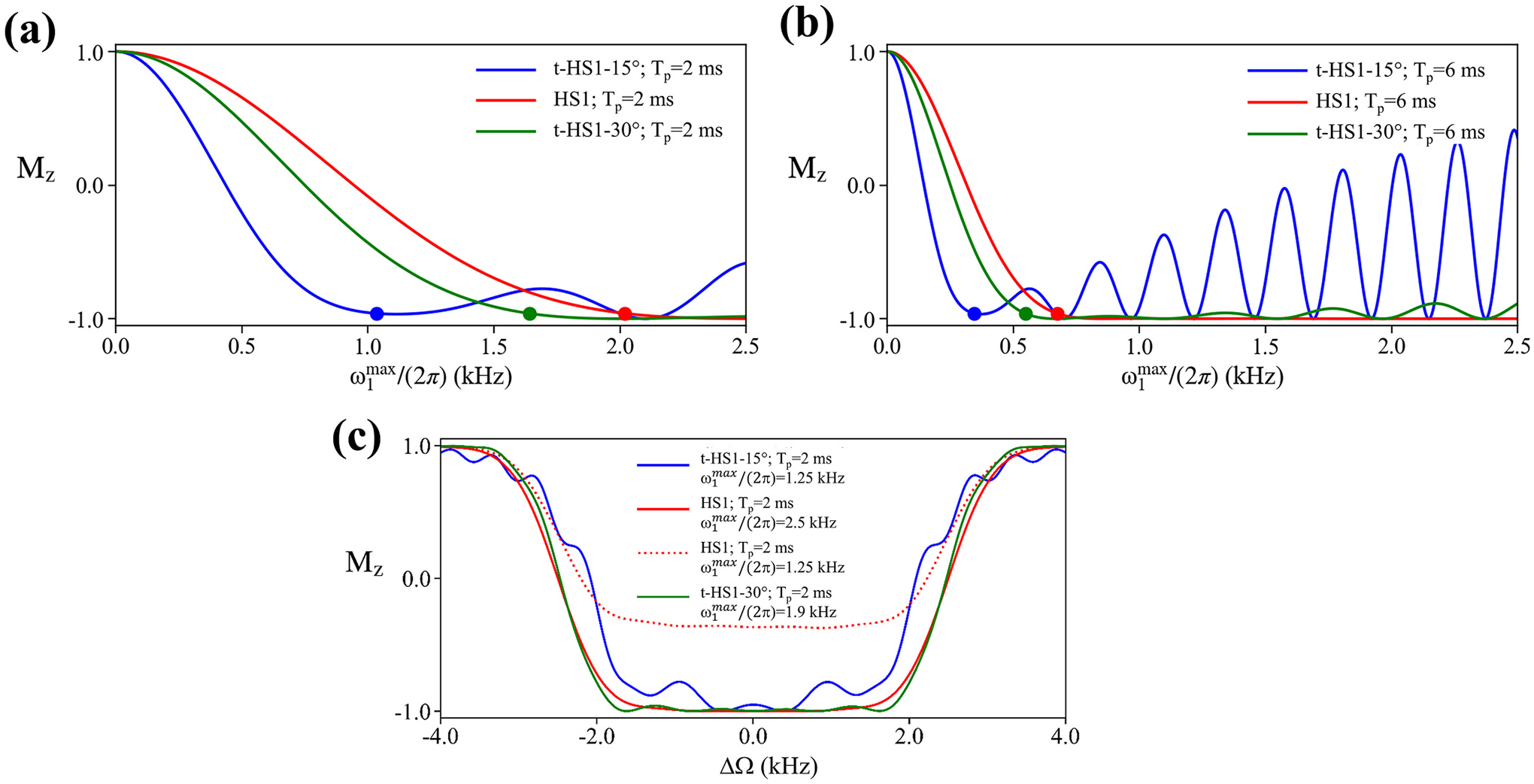
Simulations of longitudinal $M_{z}$ trajectory using Bloch equations, with no inclusion of T_1_ and T_2_ relaxation effects. (a,b) Longitudinal magnetization $M_{z}$ as a function of peak power of the HS1, t-HS1-15° and t-HS1-30° pulses at different pulse durations (T_p_ = 2 ms and T_p_ = 6 ms). Dots correspond to RF amplitudes that result in the inversion of M. (c) $M_{z}$ as a function of frequency offset (ΔΩ) for HS1, t-HS1-15° and t-HS1-30° pulses at T_p_ = 2 ms and different pulse peak amplitudes. The plots demonstrate that complete inversions are achieved with t-HS1-15° and t-HS1-30° at generally lower peak power than with HS1 (*i.e*., no truncation), albeit the profile of the inversion bandwidth is not as flat.

The dependencies of the magnetization component $M_{z}$ on B_1_ are shown in Fig. 3a for HS1, t-HS1-15° and t-HS1-30° pulses with pulse duration T_p_ = 2 ms, and in Fig. 3b with T_p_ = 6 ms. The $M_{\text{z}}$ profiles are also shown as a function of the frequency offset (ΔΩ) in Fig. 3c for T_p_ = 2 ms and different B_1_ amplitudes. As shown in Fig. 3c [44,62], the HS1 pulse with T_p_ = 2 ms requires significantly higher peak power than t-HS1-15° and t-HS1-30° pulses to achieve complete inversion of M. Furthermore, for a longer pulse duration of T_p_ = 6 ms, t-HS1-15° and t-HS1-30° pulses enable complete inversion at lower peak RF amplitudes than the HS1 pulse, a feature that is highly beneficial for *in vivo* applications.

## 5. Relaxations due to dipole-dipole interactions

The theoretical formalism of the relaxations due to homonuclear dipole-dipole interactions between like ½ spins was previously introduced for FS RF pulses [55]. Solutions for transverse and longitudinal relaxations in the WCR were derived using spectral density functions calculated in the laboratory frame, which were then transformed to high-rank rotating frames using Wigner transformations [20,37,41,55]. Solutions in the rotating frames of rank σ were obtained by performing sequential transformations between the rotating frames undergoing multi-fold rotations at $\omega_{0},\omega_{\text{eff}}^{\left(1\right)}(\text{t})\ldots \omega_{\text{eff}}^{\left(\sigma \right)}(\text{t})$. The general forms of the relaxation rate constants ${\text{R}}_{1\rho ,\sigma }(\text{t})$ and ${\text{R}}_{2\rho ,\sigma }(\text{t})$ induced by homonuclear dipolar interactions between ½ spins in the rotating frame of rank σ in a WCR are given by [55]:

(14) $$R_{1\rho ,\sigma }(t)=\frac{3}{20}\frac{\hbar^{2}\gamma^{4}}{r^{6}}\sum_{m=-2}^{2}\sum_{m^{1}=-2}^{2}\sum_{m^{2}=-2}^{2}\sum_{m^{3}=-2}^{2}\sum_{m^{4}=-2}^{2}\cdots \;\sum_{m^{\left(N-1\right)}=-2}^{2}\sum_{m^{\left(N\right)}=-2}^{2}\;J\left(m\omega_{0}+m^{1}{\omega^{\left(1\right)}}_{eff}(t)+m^{2}{\omega^{\left(2\right)}}_{eff}(t)+\sum_{\sigma =3}^{N}\left(m^{\sigma }{\omega^{\left(\sigma \right)}}_{eff}(t)\right)\right)\;{\left({d_{m,m^{1}}}^{\left(2\right)}\left(\alpha^{\left(1\right)}(t)\right)\right)}^{2}{\left({d_{m^{1},m^{2}}}^{\left(2\right)}\left(\alpha^{\left(2\right)}\right)\right)}^{2}\;\prod_{\sigma =3}^{N}{\left({d_{m^{\sigma -1},m^{\sigma }}}^{\left(2\right)}\left(\alpha^{\left(\sigma \right)}\right)\right)}^{2}{\left(m^{\sigma }\right)}^{2},$$

(15) $$R_{2\rho ,\sigma }(t)=\frac{3}{40}\frac{\hbar^{2}\gamma^{4}}{r^{6}}\sum_{m=-2}^{2}\sum_{m^{1}=-2}^{2}\sum_{m^{2}=-2}^{2}\sum_{m^{3}=-2}^{2}\sum_{m^{4}=-2}^{2}\cdots \;\sum_{m^{\left(N-1\right)}=-2}^{2}\sum_{m^{\left(N\right)}=-2}^{2}\;J\left({m\omega_{0}+m^{1}\omega^{\left(1\right)}}_{eff}\left(t\right)+{m^{2}\omega^{\left(2\right)}}_{eff}\left(t\right)+\sum_{\sigma =3}^{N}\left({m^{\sigma }\omega^{\left(\sigma \right)}}_{eff}\left(t\right)\right)\right)\;{\left({d_{m,m^{1}}}^{\left(2\right)}\left(\alpha^{\left(1\right)}(t)\right)\right)}^{2}{\left({d_{m^{1},m^{2}}}^{\left(2\right)}\left(\alpha^{\left(2\right)}\right)\right)}^{2}\;\prod_{\sigma =3}^{N}\left({\left({d_{m^{\sigma -1},m^{\sigma }}}^{\left(2\right)}\left(\alpha^{\left(\sigma \right)}\right)\right)}^{2}\right)\left(m^{\sigma }+2\right)\left(3-m^{\sigma }\right).$$

Here, the second rank reduced Wigner matrix elements are $d_{m^{\left(\sigma -1\right)},m^{\sigma }}^{\left(2\right)}\left(\alpha^{\sigma }(t)\right),\hbar$ is Planck’s constant (divided by 2π),γ is the proton gyromagnetic ratio, r is the radius between two interacting spins (in CGS units), m, m^1^, m^2^ … ${\text{m}}^{\sigma }$ are the summation indexes, $\tau_{\text{c}}$ is the rotational correlation time, and $J\left(m,m^{1},m^{2}\ldots m^{\sigma },t,\tau_{c},\omega_{0},{\omega^{\left(1\right)}}_{\text{eff}}(t),{\omega^{\left(2\right)}}_{\text{eff}}(t)\ldots \right.\left.{\omega^{\left(\sigma \right)}}_{\text{eff}}(t)\right)$ is the Lorentzian spectral density function [20,55].

Notably, Eqs. (14) and (15) not only apply when M is initially parallel or perpendicular to $B_{\text{eff}}^{\left(1\right)}(\text{t})$ in the FRF (where σ=1) and thus undergo ${\text{T}}_{1\rho }(\text{t})$ or ${\text{T}}_{2\rho }(\text{t})$ relaxations, respectively, but they also apply for any arbitrary initial orientation of M in the FRF [17], in which case relaxation is still comprised of ${\text{T}}_{1\rho }$ and ${\text{T}}_{2\rho }$ relaxation channels (Fig. 1d–f). To account for fictitious fields generated during the FS pulses, the rate constants are then calculated in the SRF (σ=2), leading to the following expressions [20,55]:

(16) $$R_{1\rho ,2dd}(t)=\frac{3}{20}\frac{\hbar^{2}\gamma^{4}}{r^{6}}\sum_{m=-2}^{2}\sum_{m^{1}=-2}^{2}\sum_{m^{2}=-2}^{2}\;J\left(m\omega_{0}+m^{1}{\omega^{\left(1\right)}}_{\text{eff}}(t)+m^{2}{\omega^{\left(2\right)}}_{\text{eff}}(t)\right)\;{\left({d_{m,m^{1}}}^{\left(2\right)}\left(\alpha^{\left(1\right)}(t)\right)\right)}^{2}{\left({d_{m^{1},m^{2}}}^{\left(2\right)}\left(\alpha^{\left(2\right)}\right)\right)}^{2}{\left(m^{2}\right)}^{2},$$

and

(17) $$R_{2\rho ,2dd}(t)=\frac{3}{40}\frac{\hbar^{2}\gamma^{4}}{r^{6}}\sum_{m=-2}^{2}\sum_{m^{1}=-2}^{2}\sum_{m^{2}=-2}^{2}\;J\left(m\omega_{0}+m^{1}{\omega^{\left(1\right)}}_{\text{eff}}(t)+m^{2}{\omega^{\left(2\right)}}_{\text{eff}}(t)\right)\;{\left({d_{m,m^{1}}}^{\left(2\right)}\left(\alpha^{\left(1\right)}(t)\right)\right)}^{2}{\left({d_{m^{1},m^{2}}}^{\left(2\right)}\left(\alpha^{\left(2\right)}\right)\right)}^{2}\left(m^{2}+2\right)\left(3-m^{2}\right).$$

An important feature of Eqs. (16), (17) is that ${\text{R}}_{1\rho ,2}(\text{t})$ and ${\text{R}}_{2\rho ,2}(\text{t})$ are functions of both angles $\alpha^{\left(1\right)}(\text{t})$ and $\alpha^{\left(2\right)}(\text{t})$. In our previous work, we demonstrated that in the WCR the relaxation rate constants in the rotating frame of rank σ could be represented through the rate constant derived in the rotating frame of rank σ−1 using trigonometric relationships between the frames [37]. Therefore, for the transverse and longitudinal rate constants in the SRF, the following trigonometric identities could be used in the WCR:

(18) $$R_{1\rho ,2\text{dd}}(t)=R_{1\rho ,1dd}(t){\text{cos}}^{2}\left(\alpha^{\left(2\right)}(t)\right)+R_{2\rho ,1dd}(t){\text{sin}}^{2}\left(\alpha^{\left(2\right)}(t)\right)$$

(19) $$R_{2\rho ,2\text{dd}}\left(t\right)=R_{2\rho ,1dd}\left(t\right){\text{cos}}^{2}\left(\alpha^{\left(2\right)}\left(t\right)\right)+R_{1\rho ,1dd}\left(t\right){\text{sin}}^{2}\left(\alpha^{\left(2\right)}\left(t\right)\right).$$

The Eqs. (18), (19) provided identical result with the analytical expressions obtained using Wigner transformations between the frames for the relaxations due to dipolar interactions during FS pulses, e.g., the RAFFn [55] and adiabatic pulses of the HSn family [17]. Others found that basic trigonometric relationships presented by Eqs. (18), (19) also apply for stationary conditions between free precession (R_1_ and R_2_) and spin-lock continuous wave (SL CW) rotating frame $R_{1\rho }$ and $R_{2\rho }$ relaxation rate constants in the Redfield relaxation limit [63–65].

## 6. Exchange-induced ${\text{R}}_{1\rho ,2\text{ex}}\;\text{and}\;{\text{R}}_{2\rho ,2\text{ex}}$ relaxations in the rotating frame of rank 2

The analytical formalism for exchange-induced relaxations between two magnetic sites, A and B, with different chemical shifts (Δω≠0) undergoing equilibrium exchange $A\underset{k_{-1}}{\overset{k_{1}}{\Leftrightarrow }}B$ had been recently presented using density matrix formalism [66]. Here, k_1_ and k_−1_ represent the forward and backward exchange rate constants fulfilling basic relationship: $\tau_{\text{ex}}=\frac{P_{A}}{k_{-1}}=\frac{P_{B}}{k_{1}}$, P_A_ and P_B_ are the intrinsic populations of the exchanging sites, $\tau_{\text{ex}}=1/{\text{k}}_{\text{ex}}$ is exchange correlation time and ${\text{k}}_{\text{ex}}={\text{k}}_{1}+{\text{k}}_{-1}$ is the rate of exchange. Previously, we derived the expressions for the longitudinal ${\text{R}}_{1\rho ,2\text{ex}}$ and transverse ${\text{R}}_{2\rho ,2\text{ex}}$ relaxations rate constants in the SRF. We have shown that the expressions are valid for description of the relaxations when the magnetization M is initially placed to any arbitrary angle $\alpha^{\left(1\right)}$ relatively to the ${\text{B}}_{\text{eff}}^{\left(1\right)}(\text{t})$, and as the specific case is the relaxations in the presence of a fictitious magnetic field generated by amplitude and frequency modulated RF pulses. The expressions were derived in the fast exchange regime (FXR), and the transformations to the SRF were used to account for the fictitious fields generated during the FS pulse. The derived expressions are as follows:

(20) $$R_{2\rho ,2\text{ex}}\left(t\right)=p_{A}p_{B}\delta \omega^{2}\;\left(\begin{array}{c} \text{cos}{\left(\alpha^{\left(2\right)}\left(t\right)\right)}^{2}\text{cos}{\left(\alpha^{\left(1\right)}\left(t\right)\right)}^{2}\tau_{\text{ex}}+\frac{1}{2}\text{sin}{\left(\alpha^{\left(2\right)}\left(t\right)\right)}^{2}\text{sin}{\left(\alpha^{\left(1\right)}\left(t\right)\right)}^{2}\tau_{\text{ex}}+\frac{1}{2}\left(\text{cos}{\left(\alpha^{\left(1\right)}\left(t\right)\right)}^{2}\text{sin}{\left(\alpha^{\left(2\right)}\left(t\right)\right)}^{2}+\frac{1}{2}\text{cos}{\left(\alpha^{\left(1\right)}\left(t\right)\right)}^{2}\text{sin}{\left(\frac{\alpha^{\left(2\right)}\left(t\right)}{2}\right)}^{4}+\frac{1}{2}\text{sin}{\left(\alpha^{\left(1\right)}\left(t\right)\right)}^{2}\text{cos}{\left(\frac{\alpha^{\left(2\right)}\left(t\right)}{2}\right)}^{4}\right)\\ \frac{\tau_{ex}}{1+{\left({\omega^{\left(2\right)}}_{eff}\left(t\right)\tau_{\text{ex}}\right)}^{2}} \end{array}\right)$$

(21) $$\begin{array}{c} R_{1\rho ,2\text{ex}}\left(t\right)=p_{A}p_{B}\delta \omega^{2}\\ \left(sin{\left(\alpha^{\left(1\right)}\left(t\right)\right)}^{2}cos{\left(\frac{\alpha^{\left(2\right)}\left(t\right)}{2}\right)}^{4}+sin{\left(\alpha^{\left(1\right)}\left(t\right)\right)}^{2}sin{\left(\frac{\alpha^{\left(2\right)}\left(t\right)}{2}\right)}^{4}+\text{cos}{\left(\alpha^{\left(1\right)}\left(t\right)\right)}^{2}\text{sin}{\left(\alpha^{\left(2\right)}\left(t\right)\right)}^{2}\right)\\ \frac{\tau_{ex}}{1+{\left({\omega^{\left(2\right)}}_{eff}\left(t\right)\tau_{ex}\right)}^{2}}. \end{array}$$

Eqs. (14)–(21) describe instantaneous rate constants at any given time during the FS pulse. The averaged ${\bar{R}}_{1,2\rho }\left(\tau_{c},\tau_{ex}\right)$ relaxation rate constant which accounts for dipolar interactions and exchange during the FS pulse of duration T_p_, incorporates all instantaneous contributions. Therefore, the effective relaxation rate constant is expressed as:

(22) $${\bar{R}}_{1,2\rho }\left(\tau_{c}\tau_{ex}\right)=\frac{1}{T_{p}}\int_{0}^{T_{p}}R_{1,2\rho ,dd}\left(\tau_{c}t\right)dt+\frac{1}{T_{p}}\int_{0}^{T_{p}}R_{1,2\rho ,ex}\left(\tau_{ex}t\right)dt.$$

Note that in Eq. 22 and in the following, the rank σ is omitted for simplicity.

## 7. Methods

### 7.1. Theoretical analyses

The theoretical calculations of dipolar and exchange-induced relaxations during the pulses presented in this work were conducted using the Mathematica 13 and 14.1 software packages. In particular, Eqs. (14)–(19) were used to evaluate relaxations due dipolar interactions, and Eqs. (20), (21) for anisochronous exchange. The effective relaxation rates during the RF pulses were obtained using Eq. (22).

### 7.2. In vivo studies

Four Sprague–Dawley rats (Envigo; males, 260–400 g) and two Wistar rats (Charles River; female, 290–300 g), were used for *in vivo* MRI relaxation measurements conducted with a 9.4 T 31-cm horizontal bore magnet (Bruker, Paravision 3.5 console, Palo Alto, CA, USA) and a quadrature volume transmit/receive coil. The studies were carried out in compliance with the ARRIVE guidelines, and experimental procedures were approved by the Institutional Animal Care and Use Committee (IACUC) of the University of Minnesota. The rats were anesthetized with isoflurane both during the preparation phase and the MRI acquisitions, with O_2_/N_2_O (30%/70%) carrier gas. In order to explore the opportunity of highlighting different relaxing components, experiments in each of the six rats were performed with both gradient recalled echo (GRE) and with MB-SWIFT readouts in the same session in randomized orders; moreover, inter-session repeatability was qualitatively assessed in 2 rats which underwent a second set of experiments with GRE readout within two weeks on average from the first session. With GRE readout the duration of the pulses was T_p_ = 6 ms, while with MB-SWIFT readout the durations of the pulses ranged from 0.7 ms to 2.0 ms [53].

### 7.3. Preparation of ex-vivo brain

In order to explore the robustness of our RETRO methodology, we measured relaxation maps with HS1 and t-HS1 pulses on an *ex-vivo* rat brain. The procedure for *ex-vivo* brain phantom preparation is detailed in Hakkarainen et al. [22]. Briefly, a healthy Sprague-Dawley rat was deeply anesthetized with isoflurane and transcardially perfused with 0.9% sodium chloride for 2 min (30 mL/min, 4C), followed by 4% paraformaldehyde in 0.1 M phosphate buffer, pH 7.4, for 20 min (30 mL/min, 4C). The fixed brain was removed from the skull and postfixed for 4 h in the same 4% paraformaldehyde solution. The brain was washed in 0.9% sodium chloride solution overnight and immersed in perfluoropolyether to avoid signal from the solution. The brain was placed tightly in a Teflon cylinder for the MRI acquisitions.

In addition, with the goal of performing correlation analyses of the MRI contrasts with tissue substrates, we used histological data published previously from the rodent brain [22]. The methods used to obtain such histological data were described previously [22]. Briefly, brains were washed in 0.9% sodium chloride, cryoprotected for 36 h in 20% glycerol (prepared with 0.02 M potassium phosphate-buffered saline), frozen on dry ice, and stored at −70 °C. Coronal sections (30μm, 1-in-5 series) were cut with a sliding microtome. The first series was stored in 10% formalin and stained with Nissl to examine cytoarchitecture. Remaining series were stored in cryoprotectant at −20 °C. The second series was stained for myelin using gold chloride, and the third series was stained for iron using Perl’s staining with 3,3′-diaminobenzidine (DAB) enhancement. Myelin, iron, and cell densities were quantified from five consecutive sections (covering 750μm) using ImageJ (v1.41o). Optical intensity was measured from ROIs in 12 brain regions, including the dentate gyrus, hippocampus, amygdala/piriform cortex, somatosensory cortex layers I and VI, thalamic nuclei, cingulum, corpus callosum, internal capsule, fimbria, optic tract, and external capsule. Optical density values were calculated by normalization of intensity from each ROI and background intensity from each section, as follows: (*mean* intensity of background–*mean* ROI intensity)/background intensity. Values were corrected for staining variability using a scaling factor based on mean ROI intensities within each section. In the correlation analyses presented in the current study, we used the 9 Waxholm atlas regions that overlapped with the 12 regions with available histological data.

### 7.4. Relaxation mapping with GRE readout in vivo

GRE readouts were implemented in 8 studies (on 6 separate rats, 2 of which underwent repeated sessions in different days) with the following parameters: echo Time (TE) = 2.58 ms, flip angle = 15°, matrix size = 128 × 128 × 13, 2 segments, segment size = 64, segment time duration = 381.33 ms; field of view (FOV) = 32 × 32 × 13 mm. For the ${\text{T}}_{1,2\rho }$ measurements, HS1 and t-HS1-15° pulses were calibrated at $\omega_{1}^{\text{max}}/(2\pi )=2.0\;\text{kHz}$ and 1.25 kHz, respectively. Both pulses were 6 ms long, with time-bandwidth product R = 10 and 9 for HS1 and t-HS1-15°, respectively. Number of pulses in the pulse train: 0, 8, 16, 24, 32 for the ${\text{T}}_{1\rho }$ and 0, 4, 8, 12, 16 for the ${\text{T}}_{2\rho }$, phase-cycled according to (0ππ0). For ${\text{T}}_{1\rho }$ acquisitions, the pulse train was placed prior to the GRE readout without further delays, while for ${\text{T}}_{2\rho }$, the pulse train was placed between an adiabatic half passage (AHP, 4 ms duration, phase 0, and peak power of 900 Hz) for coherent excitation, and a reverse AHP pulse with phase π for returning the magnetization to +z′ axis before readout. To calculate the relaxation maps, the signal intensity decays measured in each pixel during the pulse train were fitted with a custom-script 2-parameter nonlinear least-squares fitting using the Levenberg-Marquardt algorithm in Python 3.8.

### 7.5. Relaxation mapping with MB-SWIFT readout

MB-SWIFT readouts were implemented in 6 studies *in vivo* (on 6 separate rats) with following parameters: bandwidth = 125 kHz, TR = 2.297 ms, number of spokes = 4000 × 16, FOV = 40 × 40 × 40, matrix size = 128 × 128 × 128, oversampling = 2, number of gaps = 4, sideband = 256, flip angle = 3°. Adiabatic ${\text{T}}_{1\rho }$ was acquired by inserting a magnetization preparation (MP) block every n=40 readout pulses (spokes), similarly to [54]. Acquisitions were repeated with 0, 4 and 8 pulses in the MP blocks, phase-cycled according to self-compensation MLEV scheme (0,π,π,0) [67]. The HS1 pulse duration was set to T_p_ = 2 ms, while the duration of t-HS1-15° was set to 2 ms, 1 ms, and 0.7 ms. Peak power $\omega_{1}^{\text{max}}/(2\pi )$ was 2.5 kHz for the HS1 pulse, 1.25 kHz for the t-HS1-15° pulse with T_p_ = 2 ms, 2.15 kHz for the t-HS1-15° pulse with T_p_ = 1 ms, and 3 kHz for the t-HS1-15° pulse with T_p_ = 0.7 ms. To capture the short relaxation component of the SI decay, the relaxation maps were obtained by fitting the images with n=0,4,8 pulses, as detailed in Zhang et al. [54]. B_1_ and T_1_ maps were also used to estimate the relaxation maps, as described in [54]. In particular, T_1_ and B_1_ maps were acquired with alternating Look-Locker (aLL) technique [68] with the following acquisition parameters: BW = 200 kHz, repetition time TR = 1.545 ms, flip angle = 3°, each Look-Locker (LL) with k space point = 5000 × 4 (view × spiral), LL = 16, inversion pulse with HS1 R20 pulse, $\omega_{1}^{\text{max}}/(2\pi )=542\;\text{Hz}$; recovery time = 4 s, sidebands = 256; FOV = 40 × 40 × 40 mm^3^, and matrix size = 128 × 128 × 128.

For the relaxation mapping of the *ex vivo* rat brain, two phase-cycling schemes based on MLEV were implemented: (i) (00ππ) and (ii) supercycling (0ππ0) [44,67]. The pulse parameter’s settings were as follows: T_p_ = 1 ms, R = 10 and $\omega_{1}^{\text{max}}/(2\pi )=2.15\;\text{kHz}$, and the relaxation maps were obtained as for the *in vivo* studies.

### 7.6. Co-registration and relaxograms

Anatomical images were acquired for reference using a RARE sequence with the following parameters: TE = 20 ms, TR = 3423 ms, averages = 2, RARE factor = 4, matrix size = 28 × 128 × 21, FOV = 32 × 32 × 21 mm. Image co-registration procedures were performed with ANTs using a symmetric normalization (SyN) algorithm after an initial manual alignment. In particular, the relaxation maps were aligned to the corresponding anatomical reference, which was first aligned to Waxholm space atlas template [69]. The deformation fields were then combined to obtain an atlas aligned to each subject’s relaxation maps. In order to minimize partial volume and unnecessary interpolations, no smoothing was applied on the relaxation time maps.

Relaxation time constants were calculated in representative ROIs from the Waxholm atlas, and relaxograms were obtained to show the value distributions. To avoid contamination from surrounding areas, especially in small regions, outlier voxels were excluded from the calculation of average values using Tukey’s outlier detection method. Also, the 2 repeated GRE metrics from 2 rats were considered as independent variables in the group averages. Overall, a total of 8 relaxation metrics were thus computed, reflecting the adopted pulse configurations, namely: 4 metrics with GRE readout including ${\text{T}}_{1\rho }$ and ${\text{T}}_{2\rho }$ for HS1 and t-HS1-15° at T_p_ = 6 ms, and 4 metrics with MB-SWIFT readout including ${\text{T}}_{1\rho }$ of HS1 at T_p_ = 2 ms, and t-HS1-15° at T_p_ = 2 ms, 1 ms and 0.7 ms. In addition, in order to highlight the spatial diversity of the rotating frame contrasts with t-HS1 pulses, 3 relaxations rate differences $\left(\delta {\text{R}}_{1\rho }\right)$ were calculated as ${\text{R}}_{1\rho }$ of t-HS1-15° at T_p_ = 2 ms, 1 ms and 0.7 ms, minus ${\text{R}}_{1\rho }$ of HS 1 at T_p_ = 2 ms.

An ROI-based 3-dimensional representation of the relaxation maps was further achieved by first calculating the *mean* values in each ROIs of the SIGMA rat brain template [70] for each study, and then projecting the group averages on selected ROIs of the SIGMA rat template mesh using BrainNet viewer [71] in MATLAB R2019a. We then considered group average values of relaxation times from the 9 specific brain regions of the atlas for which average optical density from staining of iron, myelin and Nissl were available from a previous study [22], namely amygdala and piriform cortex (combined), corpus callosum, cingulum, dentate gyrus, fimbria, hippocampus, primary somatosensory cortex, optic tract, ventral posterior thalamic nuclei and posterior thalamic nucleus (combined). In order to establish correlations of the new relaxation contrasts with tissue components, Pearson’s coefficients were finally calculated between the 3 optical densities and 11 described relaxation metrics. Separately for each of the 3 optical densities, correlations were considered significant after Bonferroni correction for either 4 comparisons of metrics acquired with GRE readout, or 7 comparisons of metrics acquired with MB-SWIFT readout.

## 8. Results

### 8.1. Theoretical analyses

**Fig. 4.**
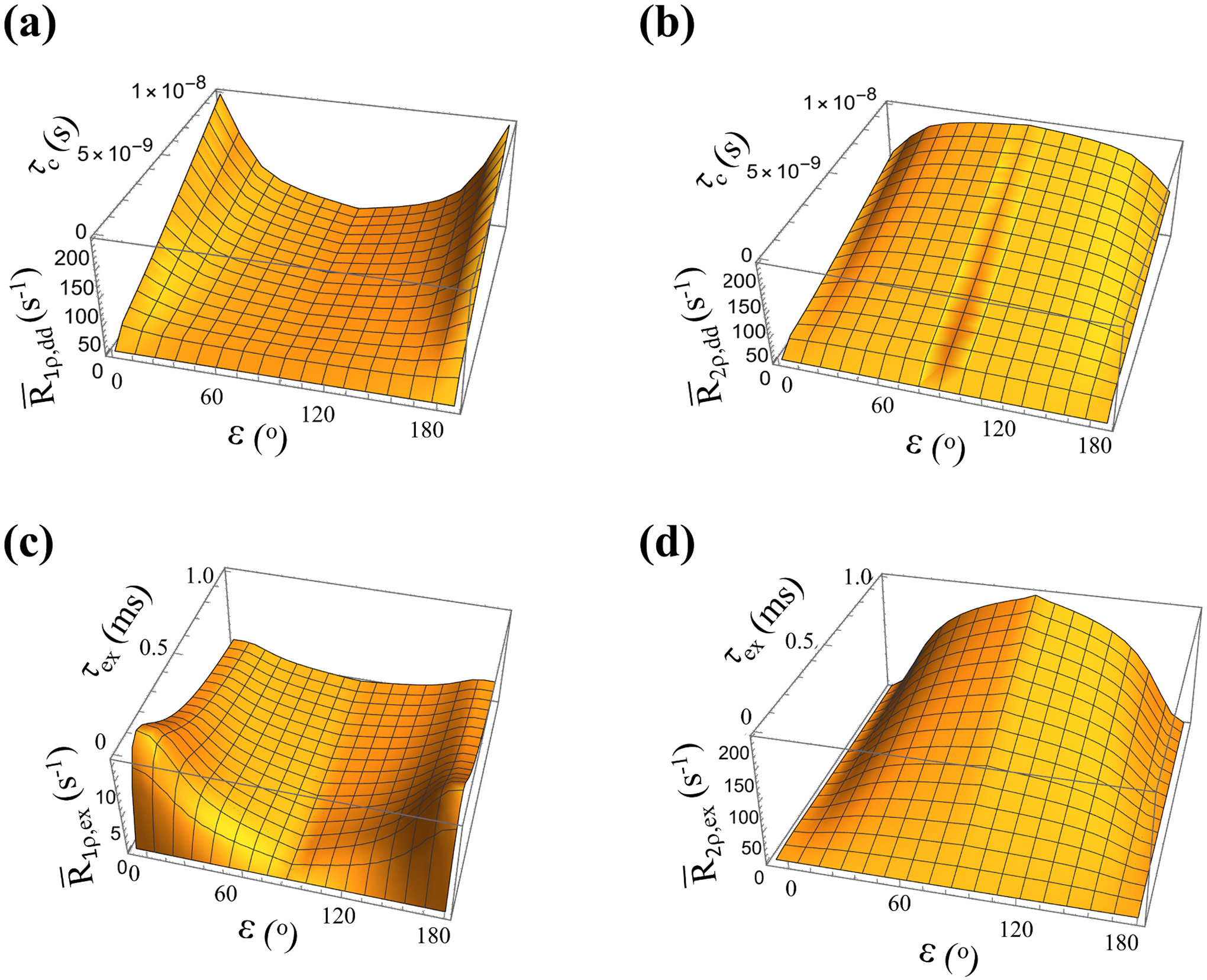
Dependence of the relaxation rates on truncation levels. (a,b) Effective relaxation rate constants ${\bar{\text{R}}}_{1\rho_{,\text{dd}}}$ and ${\bar{\text{R}}}_{2\rho_{,\text{dd}}}$ due to dipolar interactions between identical spins ½ calculated in the SRF during the t-HS1-ε pulse as a function of rotational correlation times and ε; (c,d) Exchange-induced relaxation rate constants ${\bar{\text{R}}}_{1\rho_{,\text{ex}}}$ and ${\bar{\text{R}}}_{2\rho_{,\text{ex}}}$ as a function of exchange correlation times and ε during t-HS1-ε. Pulse parameters were $\omega_{1}^{\text{max}}/(2\pi )=1.25\;\text{kHz}$, R = 10 and T_p_ = 6 ms.

The effective relaxation rate constants ${\bar{\text{R}}}_{1\rho_{,\text{dd}}}$ and ${\bar{\text{R}}}_{2\rho_{,\text{dd}}}$ due to homonuclear dipole-dipole interactions between like spins ½ calculated in the SRF during the t-HS1-ε pulse are shown in Fig. 4a,b as a function of rotational correlation times and ε. The effective exchange-induced relaxation rates ${\bar{\text{R}}}_{1\rho_{,\text{ex}}}$ and ${\bar{\text{R}}}_{2\rho_{,\text{ex}}}$ during the t-HS1-ε pulse as a function of ε and exchange correlation times are shown in Fig. 4c,d. The instantaneous rate constants during the pulses were first obtained using Eqs. (16)–(19) for dipolar relaxations and using Eqs. (20), (21) for anisochronous exchange. Subsequently, the effective rate constants were calculated by integrating the instantaneous contributions over the pulse and normalizing by the pulse duration using Eq. (22), as previously described [17,38–40]. Substantial dependence of the relaxation rate constants on the truncation level of the t-HS1 pulses is demonstrated, with ${\bar{\text{R}}}_{1\rho }$ increasing and ${\bar{\text{R}}}_{2\rho }$ decreasing when the truncation level increases. The dependence of the effective rate constants on the angle ε closely resembles the dependency of the truncation level on the angle ε (Fig. 2a). The increase of the ${\bar{\text{R}}}_{1\rho }$ occurs at larger truncation levels due to the contribution of the transverse relaxation component ${\text{R}}_{2\rho }$, while it is opposite for the ${\bar{\text{R}}}_{2\rho }$ because of the contribution of the longitudinal relaxation ${\text{R}}_{1\rho }$ that reduces the overall rate constant, as follows directly from the Eqs. (18)–(21). An increase of ${\bar{\text{R}}}_{1\rho }$ and a decrease of ${\bar{\text{R}}}_{2\rho }$ as a function of ε are evident, with substantial effects in the intervals 0<ε<45° and 135°<ε<180°, where the truncation levels are non-negligible. Such dependencies are more substantial for slow correlation times.

**Fig. 5.**
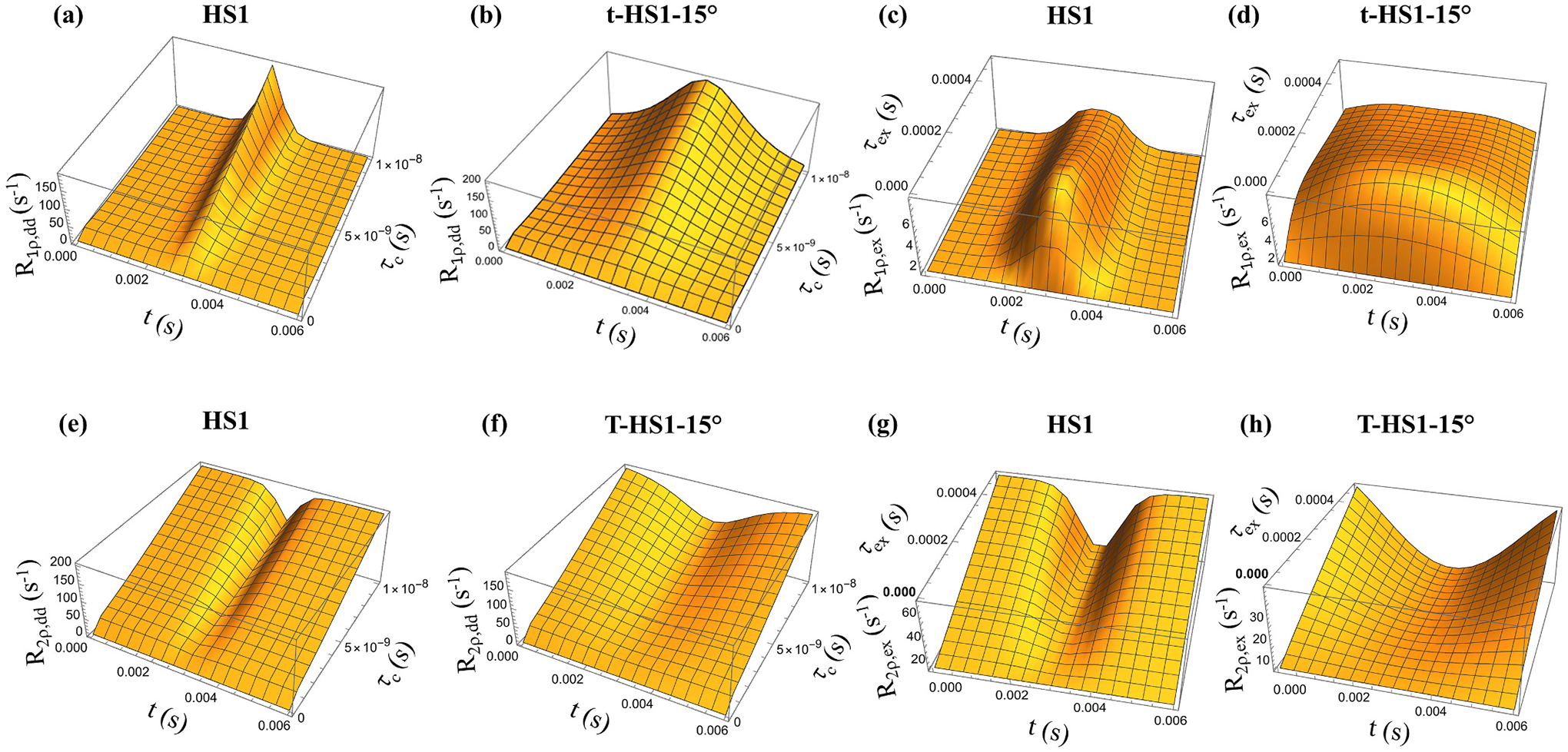
Evolution of relaxation rate contributions during the HS1 and t-HS1 pulses. (a,b) Calculated ${\text{R}}_{1\rho ,\text{dd}}$ due to dipolar interactions between like spins during the HS1 and t-HS1-15° pulses as a function of rotational correlation times (parameters as in Fig. 4); (c,d) Calculated exchange-induced ${\text{R}}_{1\rho ,\text{ex}}$ during the HS1 and t-HS1-15° pulses as a function of exchange rates. For the simulations of exchange, two sites A and B with difference in chemical shifts δω/(2π)=120Hz and P_A_ = P_B_ = 1/2 were used. (e,f) Calculated ${\text{R}}_{2\rho ,\text{dd}}$. (g,h) Calculated ${\text{R}}_{2\rho ,\text{ex}}$. Parameters were T_p_ = 6 ms, R = 10, $\omega_{1}^{\text{max}}/(2\pi )=1.25\;\text{kHz}$, and ε=45° or 15° for HS 1 and t-HS1-15°, respectively.

In Fig. 5, the instantaneous ${\text{R}}_{1\rho }$ (Fig. 5a–d) and ${\text{R}}_{2\rho }$ (Fig. 5e–h) rate constants during the pulses are shown for t-HS1-15° and HS1 pulses. In particular, the relaxation rate constants are displayed as a function of the rotational correlation times $\tau_{\text{c}}$ for the case of dipolar interactions, and of exchange rates $\tau_{\text{ex}}$ for the case of exchange. As in Fig. 4, the calculations of the rates induced by dipolar interactions were performed in the WCR using Eqs. (16)–(19), and the calculations of exchange-induced rate constants were done in the FXR using Eqs. (20), (21). Significant increase of ${\text{R}}_{1\rho }$ and decrease of ${\text{R}}_{2\rho }$ during the t-HS1-15° as compared to HS1 pulses can clearly be noticed for both exchange and dipolar relaxation mechanisms, which is due to the contributions of FRF relaxations, $R_{2\rho }$ and $R_{1\rho }$, respectively.

**Fig. 6.**
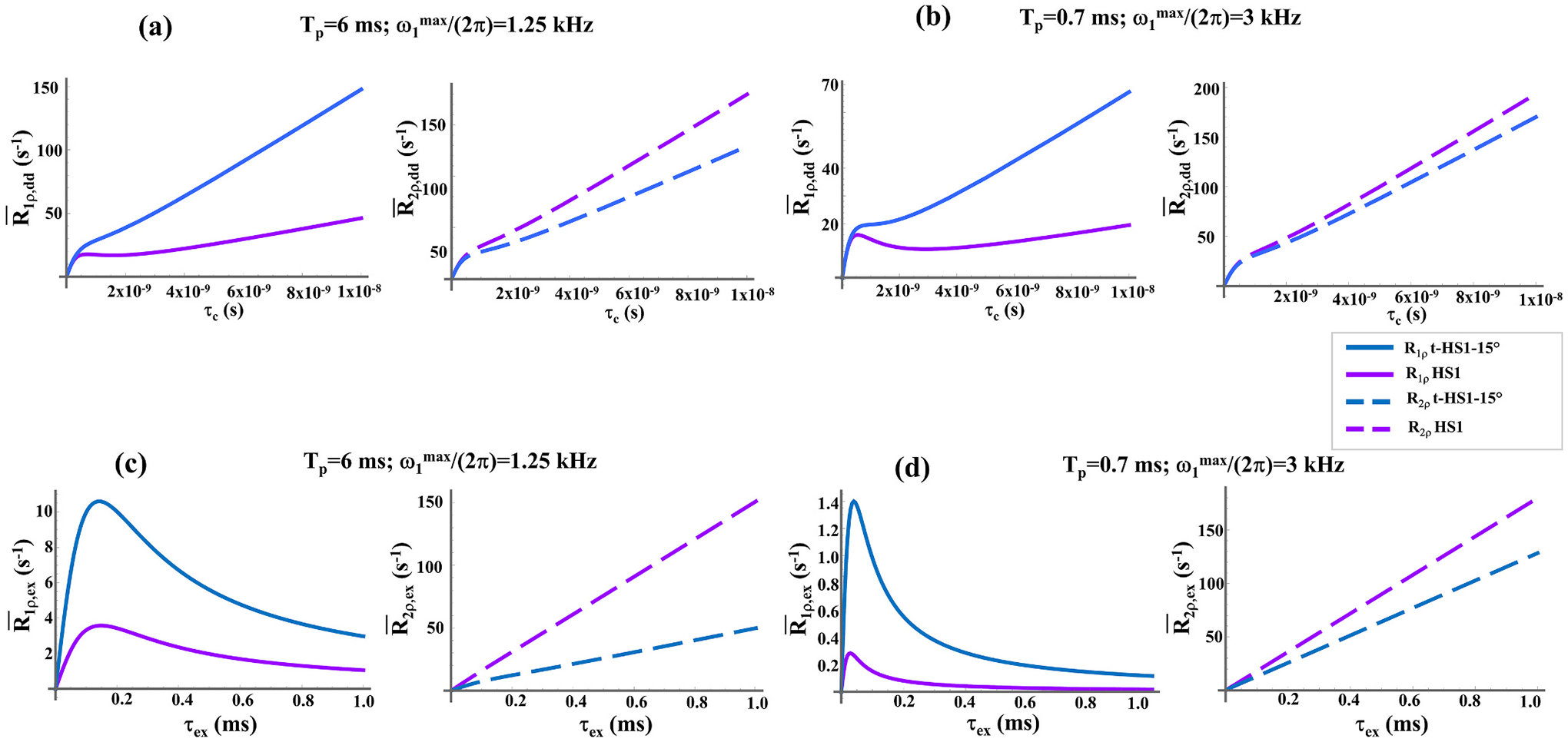
Dependencies of effective relaxation rates for different pulse durations of HS1 and t-HS1-15° pulses. (a,b) Dependencies of ${\bar{\text{R}}}_{{1\rho }_{,\text{dd}}}$ and ${\bar{\text{R}}}_{2\rho_{,\text{dd}}}$ on rotational correlation times for t-HS1-15° and HS1 pulses with pulse durations T_p_ = 6 ms (a) and T_p_ = 0.7 ms (b); (c,d) The effective exchange-induced relaxation rate constants ${\bar{\text{R}}}_{{1\rho }_{,\text{ex}}}$ and ${\bar{\text{R}}}_{{2\rho }_{,\text{ex}}}$ are shown as a function of exchange correlation times for the t-HS1-15° and HS1 pulses with pulse durations T_p_ = 6 ms (c) and T_p_ = 0.7 ms (d). For both HS1 and t-HS1-15°, pulse parameters were R = 10, while $\omega_{1}^{\text{max}}/(2\pi )=1.25\;\text{kHz}$ and 3 kHz for T_p_ = 6 ms and 0.7 ms, respectively. The instantaneous rate constants during the pulses were determined using Eqs. (16)–(21), and then integrated and normalized to the pulse duration (Eq. (22)).

The dependencies of the effective relaxation rate constants due to homonuclear dipole-dipole interactions ${\bar{\text{R}}}_{1\rho_{,\text{dd}}}$ and ${\bar{\text{R}}}_{2\rho_{,\text{dd}}}$ on the rotational correlation times for t-HS1-15° and HS1 pulses for different durations of the pulses T_p_ = 6 ms and T_p_ = 0.7 ms are presented in Fig. 6a,b, while the dependencies of effective exchange–induced relaxation rate constants ${\bar{\text{R}}}_{1\rho_{,\text{ex}}}$ and ${\bar{\text{R}}}_{2\rho_{,\text{ex}}}$ on exchange correlation times are shown in Fig. 6c,d. The plots demonstrate that ${\text{R}}_{1\rho }$ values during the t-HS1-15° are always greater than during the HS1 pulses due to the contribution of ${\text{R}}_{2\rho }$, while the opposite is true for ${\text{R}}_{2\rho }$. The observed substantial alterations of the rate constants substantiate the possibility to manipulate the relaxation contrast *in vivo* by changing the level of truncation of the FS adiabatic pulse.

### 8.2. Relaxation mapping in the rat brain

**Fig. 7.**
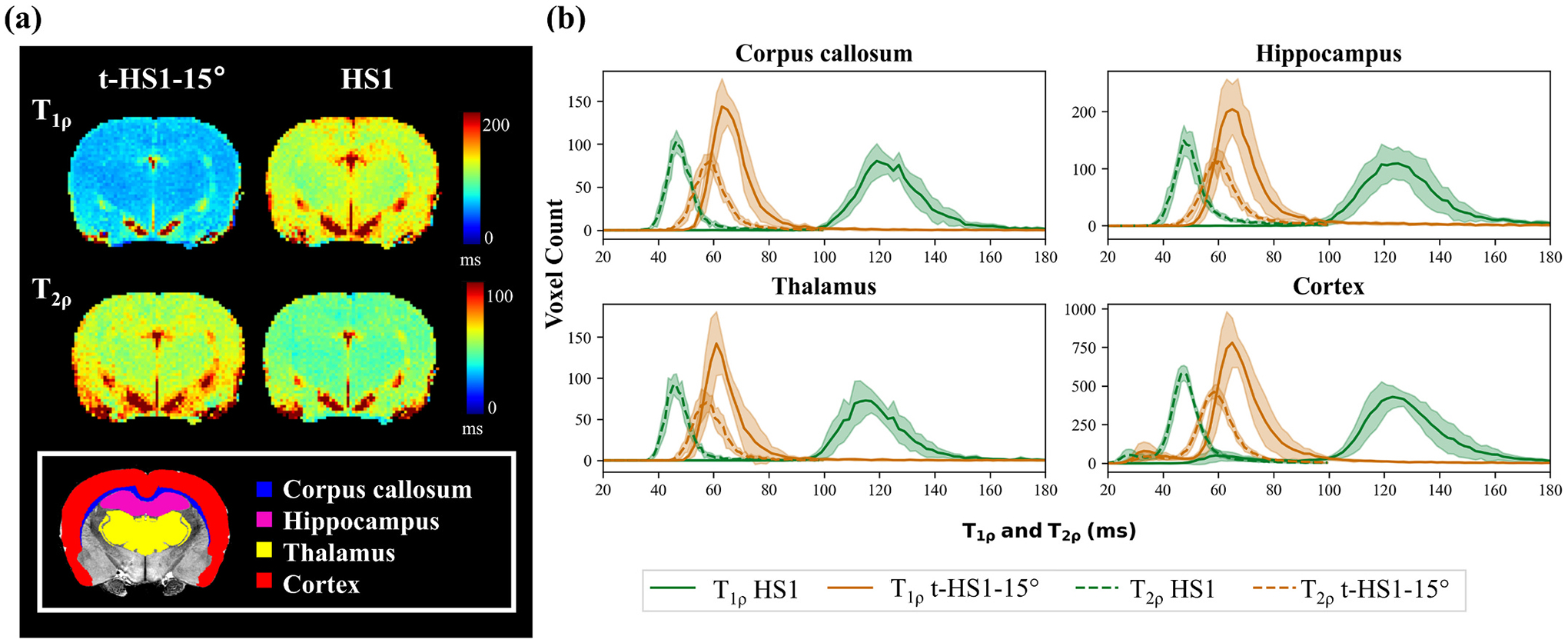
${\text{T}}_{1\rho }$ and ${\text{T}}_{2\rho }$ obtained in the rat brain using GRE readout. (a) Representative maps obtained with either HS1 or t-HS1-15° pulses. (b) Corresponding ${\text{T}}_{1\rho }$ and ${\text{T}}_{2\rho }$ relaxograms extracted from the ROIs including corpus callosum, hippocampus, thalamus and cortex (solid lines and shaded areas indicate means and SDs, respectively, across n=8 experiments- 1 for each of 6 rats plus 2 repeats). ROI masks are shown with different colors overlayed on the template of the Waxholm Space Atlas. Pulse duration was T_p_ = 6 ms for both pulses, while $\omega_{1}^{\text{max}}/(2\pi )=2\;\text{kHz}$ and 1.25 kHz for HS1 and t-HS1-15° pulses, respectively. A brain mask was applied to the maps (a) for visualization purposes.

Fig. 7a shows representative examples of ${\text{T}}_{1\rho }$ and ${\text{T}}_{2\rho }$ maps obtained with t-HS1-15° and HS1 pulses in the rat brain *in vivo* using GRE readout, along with the ROIs used to calculate the corresponding relaxograms. A summary of relaxation maps obtained in all 8 studies is given in the SF. 1, visually confirming reproducibility of the maps. As predicted by the theoretical calculations shown in Figs. 5 and 6, the relaxograms shown in Fig. 7b highlight shorter ${\text{T}}_{1\rho }$ in all ROIs with the t-HS1-15° pulse as compared to that obtained with HS1, and oppositely, longer ${\text{T}}_{2\rho }$ with the HS1 pulse as compared to t-HS1-15°. Relative differences in relaxation time constants obtained with t-HS1 *vs* HS1 were generally larger for ${\text{T}}_{1\rho }$ (about −50%) than for ${\text{T}}_{2\rho }$ (about +30%).

**Fig. 8.**
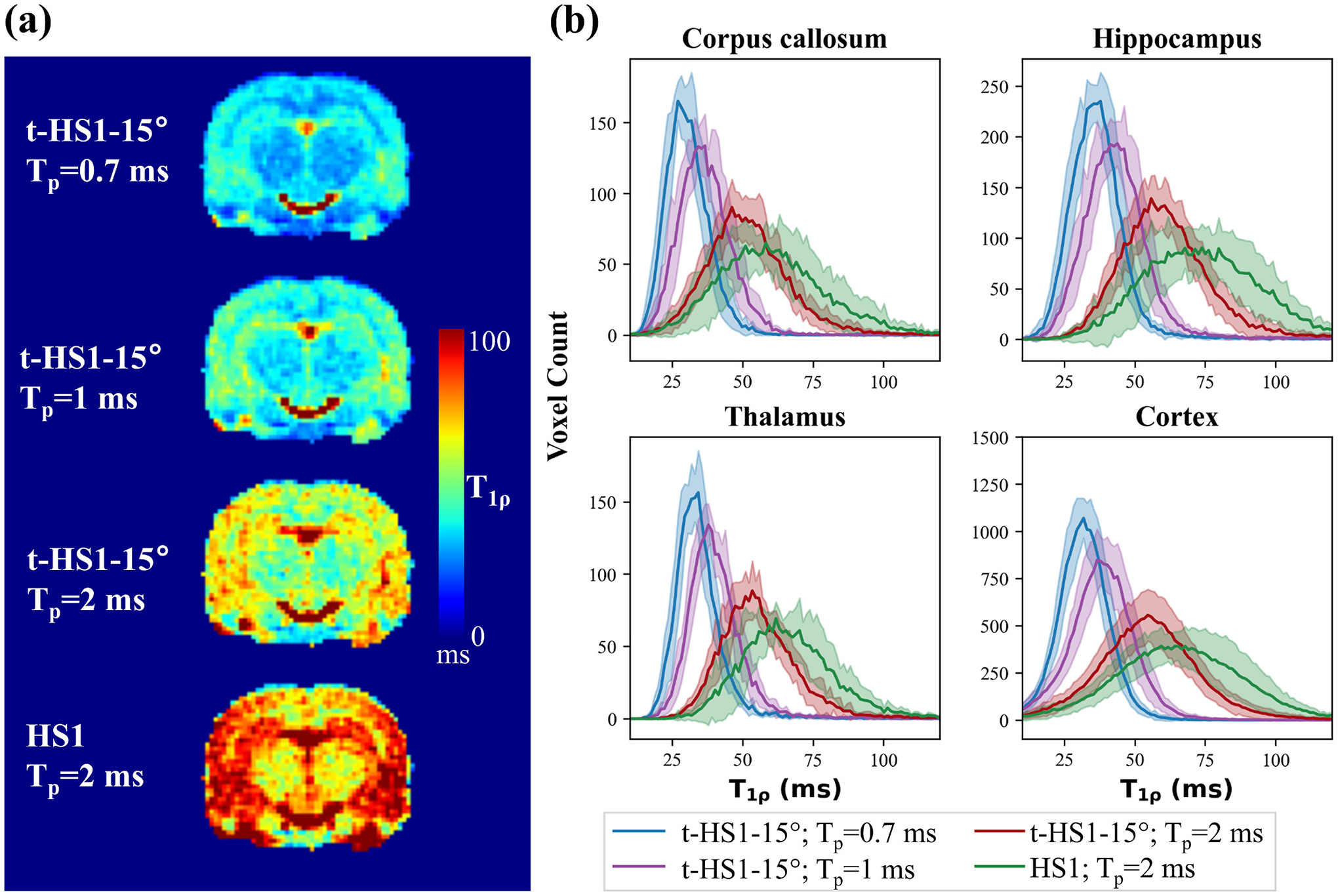
${\text{T}}_{1\rho }$ values obtained in the rat brain using zero-TE MB-SWIFT readout. (a) ${\text{T}}_{1\rho }$ maps obtained with 4 different pulse configurations in one representative rat; (b) Relaxograms averaged across 6 rats, extracted from the ROIs including corpus callosum, hippocampus, thalamus and cortex (solid lines and shaded areas indicate means and SDs, respectively, across n=6 experiments, 1 for each of 6 rats). For visualization purposes the maps have been spatially smoothed using FSL’s SUSAN noise reduction filter with a 1.16 mm kernel. Pulse configurations were: HS1 pulse with T_p_ = 2 ms and $\omega_{1}^{\text{max}}/(2\pi )=2.5\;\text{kHz}$; t-HS1-15° with T_p_ = 2 ms and $\omega_{1}^{\text{max}}/(2\pi )=1.25\;\text{kHz}$; t-HS1-15° with T_p_ = 1 ms and $\omega_{1}^{\text{max}}/(2\pi )=2.15\;\text{kHz}$; t-HS1-15° with T_p_ = 0.7 ms and $\omega_{1}^{\text{max}}/(2\pi )=3\;\text{kHz}$. A brain mask was applied to the maps (a) for visualization purposes.

Examples of ${\text{T}}_{1\rho }$ maps obtained with zero-TE MB-SWIFT readout in combination with HS1 and t-HS1-15° at various pulse durations are shown in Fig. 8a, while the ROI relaxograms averaged across subjects are displayed in Fig. 8b. In SF. 2, we present individual relaxation maps from the 6 rats, visually confirming ‘reproducibility of the maps. Substantial ${\text{T}}_{1\rho }$ differences were observed across pulses in all ROIs. Again, consistent with the theoretical predictions, t-HS1-15° ${\text{T}}_{1\rho }$ relaxation time constants were shorter than ${\text{T}}_{1\rho }$ values obtained with HS1 pulse of the same duration, and they further decreased with shorter t-HS1-15° pulses.

**Fig. 9.**
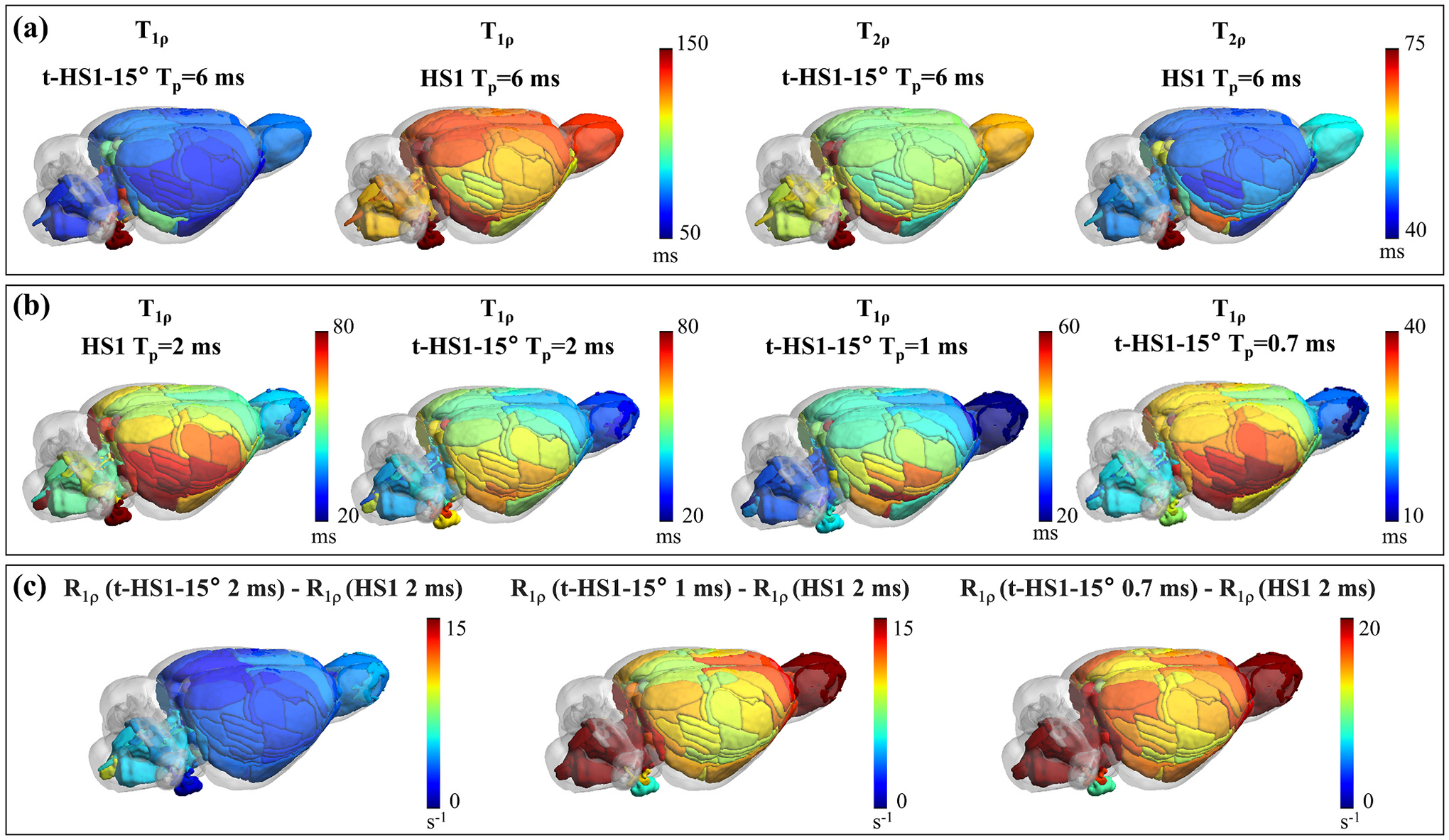
3D representations of relaxation time constants in the rat brain. Three-dimensional representation of the ${\text{T}}_{1\rho }$ and ${\text{T}}_{2\rho }$ relaxation time constants averaged across rats in selected ROIs from the SIGMA rat atlas (n = 6 for T_p_ = 6 ms and n=8 for other T_p_s). (a) ${\text{T}}_{1\rho }$ and ${\text{T}}_{2\rho }$ were measured with HS1 and t-HS1-15° pulses using T_p_ = 6 ms. GRE was used as a readout. For the pulses T_p_ = 6 ms were used. Cerebellum has been excluded from the considered ROIs due to unreliable coverage across experiments; (b,c) Three-dimensional representation of the averaged across rats ${\text{T}}_{1\rho }$ relaxation time constants (b) and the differences ${\text{R}}_{1\rho }=1/{\text{T}}_{1\rho }$ of relaxation rates obtained with different pulse durations (c). The durations of the pulses were 0.7, 1 and 2 ms. ${\text{R}}_{1\rho }$ measurements were performed with HS1 and t-HS1-15° pulses with MB-SWIFT imaging readout.

A 3D representation of relaxation contrasts obtained with t-HS1 and HS1 pulses is provided in Fig. 9. In particular, Fig. 9a summarizes the ${\text{T}}_{1\rho }$ and ${\text{T}}_{2\rho }$ maps for t-HS1-15° and HS1 pulses with T_p_ = 6 ms using GRE readout, while Fig. 9b displays the ${\text{T}}_{1\rho }$ maps obtained with MB-SWIFT readout and pulse durations T_p_ = 2, 1 and 0.7 ms. The figure demonstrates substantial difference between relaxation times detected with HS1 and t-HS1-15° pulses, as well as a significant dependency of the relaxation times on the duration of the pulses. The ${\text{R}}_{1\rho }$ differences obtained between different t-HS1-15° pulses with different pulse durations and the HS1 pulse using MB-SWIFT readout are shown in Fig. 9c. The greatest spatial contrast across white matter and gray matter regions was observed in the difference maps between ${\text{R}}_{1\rho }$ (t-HS1-15°) T_p_ = 0.7 ms and ${\text{R}}_{1\rho }$ (HS1) T_p_ = 2 ms. These observations highlight the enhanced sensitivity of t-HS1 to slow tumbling spins (*i.e*., fast relaxations) when used with short pulse durations in combination with zero-TE readouts.

Spatial distribution of ${\text{T}}_{1,2\rho }$ with different pulse configurations was also correlated with optical density distributions of myelin, iron and Nissl as measured in rats from a previous study [22]. Results of the correlation analysis are presented in Table 1. Notably, the highest statistically significant correlations with myelin were observed with $\delta {\text{R}}_{1\rho }$, obtained as a difference between t-HS1-15° with T_p_ = 1 ms and t-HS1-15° and HS1 pulses with the durations T_p_ = 2 ms.

**Fig. 10.**
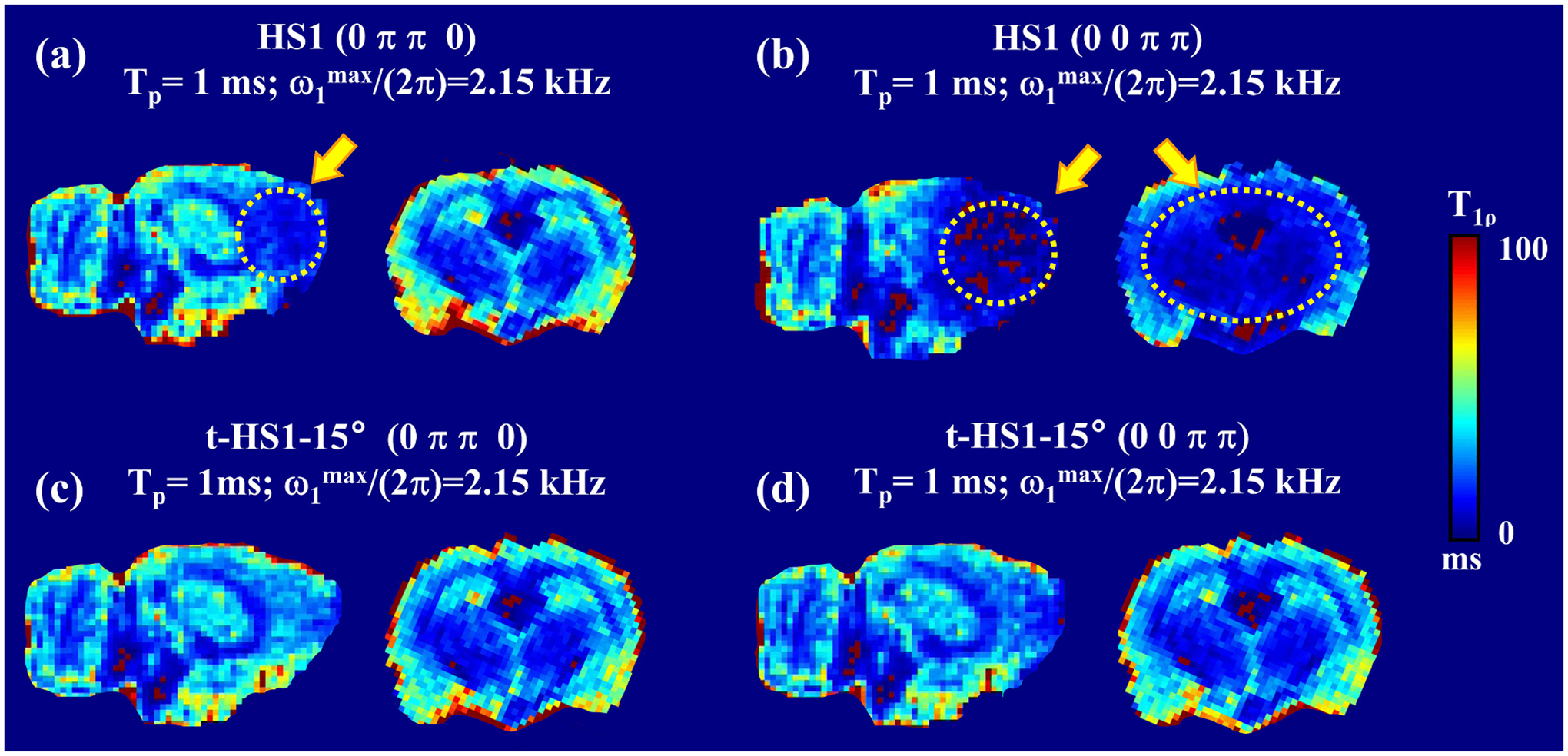
${\text{T}}_{1\rho }$ maps in sagittal and transverse orientations obtained with HS1 and t-HS1-15° pulses from the ex vivo rat brain. For comparison, two MLEV4 phase cycling schemes were implemented: (00ππ) and the supercycle with phase-inverted counterparts (0ππ0). The non-refocused artifacts are indicated by arrows and yellow outlines. The pulse parameters were as follows: T_p_ = 1 ms, RR=10 and $\omega_{1}^{\text{max}}/(2\pi )=2.15\;\text{kHz}$. Maps were manually masked to highlight the *ex vivo* brain.

Finally, to highlight the benefits of t-HS1 pulses *vs* HS1 pulse configurations when short pulse durations are desirable but safety limits may constrain the allowable RF power, we show ${\text{T}}_{1\rho }$ relaxation maps from the *ex vivo* rat brain obtained using MB-SWIFT readout with HS1 and t-HS1-15° pulses (Fig. 10). Both pulses were set at T_p_ = 1 ms and $\omega_{1}^{\text{max}}/(2\pi )=2.15\;\text{kHz}$, and pulse trains were phase-cycled according to either an MLEV4 scheme (00ππ) or a supercycle scheme with phase-inverted counterparts MLEV4 (0ππ0) (for details, see Levitt et al. [67,72]) for reducing image artifacts that may result from imperfect inversions. The pulse parameters were chosen as an example of a plausible scenario for *in vivo* applications, where the power is sufficient to obtain inversion with the t-HS1-15° pulse despite its short duration, but not with HS1. The figure demonstrates a pronounced advantage of using the t-HS1-15° pulse compared to HS1 when both phase-cycling schemes were utilized. Specifically, significant B_1_ artifacts were observed with the HS1 pulse (Fig. 10a,b) in the acquisition using phase-cycling (00ππ) and, to a lesser extent, with the phase-cycling scheme (0ππ0); meanwhile, the artifacts were refocused when using t-HS1-15° pulses with the same phase-cycling strategies (Fig. 10c, d).

## 9. Discussion

Tissue relaxations processes *in vivo* comprise contributions from multiple pathways that involve complex spin dynamics in a broad motional regime, from ultra-slow to ultra-fast motions. When conventional quantitative MRI modalities are used separately, they are insufficient to adequately address the complexity of tissue relaxation pathways, corroborating the use of multimodal approaches for characterization of the dynamic motional parameters *in vivo* [73]. Complete characterization of the proton spin dynamics in tissue requires extension of the theoretical formalism of tissue relaxations towards slow/ultraslow motional regimes where the conventional Bloembergen, Purcell and Pound theory is inadequate [74]. For instance, Yablonskiy et al. recently introduced an advanced Transient Hydrogen Bond (THB) model that describes a mechanism responsible for anisotropy of free precession relaxations in tissue through dipole-dipole interactions [49], and included a stretched spectral density function to correct the commonly used Lorentzian spectral density. On the other hand, it had been long recognized that spin dynamics could be assessed in more details with rotating frame relaxation experiments [15] as compared to the free precession T_1_ and T_2_, which supplement the arsenal of quantitative MRI methods used at high magnetic fields available through conventional free precession T_1_ or T_2_ -based methodologies [75].

In this work a multi-pronged strategy towards exploring novel MRI contrasts sensitive to slow dynamics *in vivo* was employed. The theoretical modelling of the relaxations induced by homonuclear dipolar interactions between like spins ½ and anisochronous exchange in the rotating frame of rank σ=2 allowed to describe the contributions of both ${\text{T}}_{1\rho }$ and ${\text{T}}_{2\rho }$ relaxation pathways to the relaxations detected with t-HS1-ε pulses defined by Eqs. (3) and (4). We have shown that, by changing the truncation level of the conventional hyperbolic secant pulse, one can alter the relative contributions of the ${\text{T}}_{1\rho }$ and ${\text{T}}_{2\rho }$ relaxations during the application of the pulse. This allows to manipulate relaxations and to generate tissue contrasts in MRI with high flexibility. Importantly, large level of truncation of the HS pulse enables reduction of the duration of the pulses (reducing energy deposition) while still using peak RF amplitude suitable for *in vivo* quantitative rotating frame MRI. In conjunction with the zero-TE MB-SWIFT imaging readout, the ultra-short t-HS1-15° pulses with the millisecond and less pulse durations allowed to capture fast relaxing spins of the inhomogeneously broadened tissue line, which is not possible to detect *in vivo* with conventional free precession, spin-lock continuous wave or adiabatic rotating frame relaxation techniques.

To evaluate the utility of t-HS1 pulses for tissue characterization, we performed correlation analysis between the spatial distribution of ${\text{R}}_{1,2\rho }$ and $\delta {\text{R}}_{1\rho }$ with that of optical density of myelin, iron and Nissl staining available in the rat brain from the work by Hakkarainen et al. [22]. Our results demonstrate highest correlations between myelin density and rate constant differences, $\delta {\text{R}}_{1\rho }$, between shortest (T_p_ = 0.7 and 1 ms) t-HS1-15° pulses and HS1 pulse at T_p_ = 2 ms (Table 1). This observation may be attributed to the different dependencies of ${\text{R}}_{1\rho \text{,dd}}$ due to dipole-dipole relaxation and ${\text{R}}_{1\rho ,\text{ex}}$ induced by anisochronous exchange on pulse durations T_p_ (Fig. 6), where greater change of $R_{1\rho ,\text{ex}}$ with the duration of the pulses as compared to ${\text{R}}_{1\rho \text{,dd}}$ could be noticed. Thus, upon subtraction of the rate constants detected with different pulse durations the remaining contribution to the $\delta {\text{R}}_{1\rho }$ could arise from anisochronous exchange. Yet, detailed quantitative assessment of the observed correlations requires considerations of other mechanisms, such as cross-relaxations or isochronous exchange, which are outside of the scope of current presentation. Furthermore, other combinations of the ${\text{R}}_{1\rho }$ and ${\text{R}}_{2\rho }$ obtained with different pulse parameters, which may selectively correlate with Nissl and iron stainings, i.e., measurements of neural density and iron accumulation, respectively, warrant further evaluation.

The distinct advantage of using truncated HS1 pulses for rotating frame mapping is demonstrated in Fig. 10 that shows ${\text{T}}_{1\rho }$ maps obtained with the HS1 and t-HS1-15° pulses. In Fig. 10a,b, the ${\text{T}}_{1\rho }$ maps were obtained using HS1 pulses operating in non-adiabatic regime, where the power used for the pulses is insufficient for complete inversion of magnetization M. Note that in the non-adiabatic regime, the magnetization M, if initially unperturbed and oriented along z′-axis, is not locked along $B_{\text{eff}}\left(\text{t}\right)$ during the inversion process. To measure specific relaxation pathways we embarked on comparison between two distinct phase cycling strategies with MLEV4 (00ππ) and (0ππ0), the supercycle with phase-inverted counterparts [67]. Fig. 10b demonstrates that the (0ππ0) phase cycling resulted in better quality of maps with smaller artifacts compared to the (00ππ). This observation can be attributed to more efficient refocusing during the pulse trains because it is comprised of two δ-cycles, (0π) and (π0). The (0ππ0) phase cycling was demonstrated to be highly effective for compensating RF pulse imperfections, B_1_ inhomogeneities, and frequency offsets arising from B_0_ variations and magnetic susceptibility differences in the sample, and its principles were also utilized in designing composite pulses, such as BIR-4 and RAFFn pulses [19–21,44]. Notably, during the application of t-HS1-15°, the magnetization M when initially oriented along z′ axis is also not locked along $B_{\text{eff}}\left(\text{t}\right)$. In contrast to the HS1 pulse, however, the t-HS1-15° enabled complete inversion of M at the same power level used for HS1 pulse, i.e., $\omega_{1}^{\text{max}}/\left(2\pi \right)=2.15\;\text{kHz}$, and provided artifact-free relaxation maps for both phase-cycling strategies (Fig. 10c,d).

Tissue contrast *in vivo* could originate from multiple contributing dynamic factors, including global tumbling of macromolecules, buried water and transient hydrogen bonding [49,75]. All of these components may directly or indirectly reflect a slow motional component of the spectral density, which may be associated with macromolecules that, due to molecular weight and anchoring to cellular structures, have limited mobility, *e.g*., collagen or glycogen. Regional distribution of these macromolecules varies substantially *in vivo* [76] and may be critical for characterizing brain metabolism [77]. For example, glycogen is a glucose polysaccharide consisting of approximately linear 8 to 12 alpha − 1,4 linked units that terminate with alpha 1,6 linkages forming branches. Due to branching, the molecule can have up to 11–12 tiers and a molecular weight of 10^7^ to 10^8^ Da. Despite the extremely large size, in ^13^C MRS the resonances have T_2_ values on the order of 5 to 10 ms and all visible *in vivo* and *in vitro* at 37 °C. The longer than anticipated T_2_ and overall visibility can be modeled as due to longer $\tau_{\text{c}}$ values of the motion of the individual 8–12 glucose units. The longer $\tau_{\text{c}}$ dominates T_1_ relaxation and partially averages out the rapid T_2_ relaxation anticipated due to the particles high molecular weight [78]. ^13^C MRS has relatively low sensitivity and requires ^13^C labeling to measure glycogen in the brain [79]. Ideally, to study the dynamic role of glycogen as a rapid fuel source for supporting brain activation [77,80], a more sensitive ^1^H MRS or H_2_O exchange based MRI method would be desirable.

Paradoxically, although complete ^1^H MRS visibility has been shown in solution with average relaxation rates consistent with those found using ^13^C MRS [78], only partial visibility has been reported *in vivo* despite multiple approaches such as ^1^H MRS, Nuclear Overhauser Enhancement (NOE) [81,82] or Chemical Exchange Saturation Transfer (CEST) [83]. We hypothesize that the loss of visibility is due to multiple additional slow motional relaxation pathways present *in vivo* resulting from both the heterogeneity of particle size and, more importantly, the variation of relaxation pathways caused by anchoring of particles to glycogen in and the outer tiers bound non covalently to the large macromolecular enzymes glycogen phosphorylase and synthase [84]. Indeed, even in phantoms, off- resonance spin-lock ${\text{T}}_{1\rho }$ experiments have concluded that ${\text{T}}_{1\rho }$ relaxation dispersion cannot be interpreted by a spectral density function with a single correlation time [85], manifesting the presence of spin dynamics in fast and slow regimes of motions [86]. Such premises, along with the flexibility of t-HS1 pulses to be sensitized to slow motion, warrant further investigations to determine whether t-HS1 pulses could provide a tool for non-invasive detection of glycogen, for instance in animal models with known alterations in glycogen content [87]. Overall, we anticipate that the early observations made in the current study could pave the way for the RETRO technique to be established as a viable modality for detecting fast relaxing spin packets *in vivo*, and thus RETRO may find applications in preclinical and clinical evaluations of, *e.g*., glycogen storage diseases [87].

Previous reports from our laboratory demonstrated that adiabatic ${\text{T}}_{1\rho }$ and ${\text{T}}_{2\rho }$ techniques are predominantly sensitive to spin dynamics in the μs time scale, while the sensitivity of the relaxation methods based on fictitious fields, such as RAFFn [19], is shifted to the millisecond time scale [23]. With the development of RETRO technique in the present study, we achieved manipulation of the MRI contrast by varying the truncation level of the modulation functions of the HS pulse, a choice that allowed reducing pulse durations to a sub-ms time scale and still achieve complete inversion of magnetization with RF peak powers sufficiently for *in vivo* studies. The RETRO approach inherently comprises ${\text{T}}_{1\rho }$ and ${\text{T}}_{2\rho }$ relaxation mechanisms, providing the capability to tune the sensitivity of quantitative MRI to slow motions by expanding the range of the effective frequencies to fast relaxing spins for ${\text{T}}_{1\rho }$ acquisitions and to slower relaxations with ${\text{T}}_{2\rho }$ detection. Our theoretical predictions of dipolar relaxations and exchange, although presented in the WCR and FXR only, and largely limited to the most parsimonious models of homonuclear dipole-dipole interactions between like ½ spins and fast anisochronous exchange (Figs. 4–6), were able to qualitatively describe the trends of relaxation contrasts *in vivo* observed with HS pulse with high level of truncation (Figs. 7–9 and SFs. 1 and 2). The theoretical formalism recently developed by Sorce et al. for dipolar interactions in the intermediate and strong collision regimes (ICR and SCR) during AM and FM RF pulses could be essential for the analysis of relaxation contrast observed in MRI [86]. Notably, the current development is of particular relevance for analytical solutions of Bloch equations of the HS and chirp pulses [88], and for evaluating MRI contrasts based on Rabi frequencies produced in high-rank rotating frames by spatially-selective spin inversion based on the B_1_ gradients [89]. Future research could be focused on altering the truncation levels using different functional dependencies of a broad arsenal of AM and FM modulated pulses.

Notably, the combined ${\text{T}}_{1\rho }$ and ${\text{T}}_{2\rho }$ contributions to the relaxations could be accomplished with different strategies. These strategies include AM/FM modulated pulses with the modulation functions other than HS pulse, as well as CW RF irradiation. In the case of CW irradiation, the mismatch of magnetization M with the effective magnetic field for resonances far from the carrier frequency results in oscillatory behavior of M.

Conventionally, off-resonance CW ${\text{T}}_{1\rho }$ experiments are conducted by rotating the magnetization M (often adiabatically) to a certain angle and then locking it along the $B_{\text{eff}}^{\left(1\right)}$ using a CW spin-lock pulse [90]. However, if M is not perfectly locked along the $B_{\text{eff}}^{\left(1\right)}$, then both ${\text{T}}_{1\rho ,1}$ and ${\text{T}}_{2\rho ,1}$ relaxation pathways contribute, and M undergoes precession on a cone along the direction of $B_{\text{eff}}^{\left(1\right)}$. In this case, the components of magnetization are described by modified Bloch equations, where the component of M oriented along $B_{\text{eff}}^{\left(1\right)}$ relaxes with time constant ${\text{T}}_{1\rho ,1}$, while the components oriented in the plane perpendicular to $B_{\text{eff}}^{\left(1\right)}$ relax with time constant ${\text{T}}_{2\rho ,1}$ [45]. Combined ${\text{T}}_{1\rho }$ and ${\text{T}}_{2\rho }$ relaxations can occur in the case of large B_0_ inhomogeneities, resulting in imperfect spin-locking and the formation of image artifacts [91]. This scenario has been addressed in detail in multiple contributions [15,91–93]. This complex magnetization behavior can be mitigated by using spoiler gradients to eliminate unwanted transverse magnetization. The advantage of the RETRO method is that the artifacts originating from imperfect inversion of $M_{z}$ during the FS t-HS-ε pulses could be completely eliminated by MLEV 4 cycling (0,π,π,0) while still preserving sufficiently large BW of the pulses.

During the application of an adiabatic pulse, if magnetization M is initially oriented along an arbitrary angle relatively to z′ of FRF, the relaxation process is comprised of ${\text{T}}_{1\rho ,1}(\text{t})$ and ${\text{T}}_{2\rho ,1}(\text{t})$ relaxation pathways [37]. We have also shown that during non-adiabatic RAFFn technique with *Sine* amplitude and *Cosine* frequency modulation functions, the relaxations are composed of ${\text{T}}_{1\rho ,1}(\text{t})$ and ${\text{T}}_{2\rho ,1}(\text{t})$ channels due to the formation of fictitious magnetic fields in the multifold rotating frames of rank n [19–21]. For RAFF2 with the orientation of the fictitious field at $\alpha^{\left(2\right)}=45{}^{\circ}$, the contributions of ${\text{T}}_{1\rho ,1}$ and ${\text{T}}_{2\rho ,1}$ are equal, while it is unequal for different $\alpha^{\left(2\right)}$ angles [21]. In addition, their relative ratio depends on the rank of the rotating frame. When the arbitrary angle λ that defines the initial orientation M relatively to z′ axis of the FRF is set to zero, both ${\text{R}}_{1\rho ,1}(\text{t})$ and ${\text{R}}_{2\rho ,1}(\text{t})$ contribute to the relaxation processes [66]. Only when $\lambda =\alpha^{\left(2\right)}$,the relaxation is governed solely by ${\text{R}}_{1\rho ,2}$ mechanisms, and when $\lambda =90{}^{\circ}-\alpha^{\left(2\right)},M$ undergoes precession in the plane perpendicular to ${\text{B}}_{\text{eff}}^{\left(2\right)}(\text{t})$, and thus the relaxation is governed by ${\text{R}}_{2\rho ,2}$. Although in the current development we limit the description of the relaxation contrasts generated based on different ${\text{T}}_{1\rho ,1}$ and ${\text{T}}_{2\rho ,1}$ contributions during the HS1 pulses with different levels of truncations, similar considerations are applicable for a broad family of adiabatic pulses with other modulation functions [44].

We note that the strategy for analyzing the effects of combined ${\text{T}}_{1\rho }$ and ${\text{T}}_{2\rho }$ weightings on relaxation contrast based on varying the truncation level of the HS1 pulses is novel. While obtaining combined ${\text{T}}_{1\rho }$ and ${\text{T}}_{2\rho }$ weightings to the relaxations is not new in MRI, and several approaches were proposed for altering the relative contributions ${\text{T}}_{1\rho }$ and ${\text{T}}_{2\rho }$ during FS pulses (for example, see [19,20]), the proposed methodology of changing the truncation level offers unique advantages. Notably, altering the relative ${\text{T}}_{1\rho }$ and ${\text{T}}_{2\rho }$ contributions while reducing the peak power and the pulse durations of the FS pulses promises high applicability for RETRO in studies of healthy and diseased states.

Several limitations of this study should be highlighted here. Because this work is primarily meant to introduce the underlying concept of the RETRO method, we focused our experimental studies on just one configuration of the pulses, namely ε=15°. Relaxation dispersion studies as a function of ε will be a subject of future investigations. The theoretical treatment of the dipole-dipole relaxations and anisochronous exchange provided herein addresses plausible relaxation mechanisms in tissue related to water protons in the fast motional regime. It is not meant to provide a complete description of tissue contrasts generated non-invasively with the RETRO technique, particularly in the intermediate and slow regimes. In fact, other important relaxation channels should be considered, *e.g*., diffusion in local susceptibility gradients and cross relaxations. The novel non-invasive contrast introduced in this work promises various applications as it could be tuned to different molecular motional regimes. However, validation studies using transgenic animal models with known induced pathology are warranted to determine the sensitivity and specificity of RETRO to the biological substrates of interest, potentially including, but not limited to, glycogen.

## 10. Conclusions

A new method entitled RETRO, which allows contrast manipulation by altering the truncation level of the amplitude- and frequency-modulated RF pulses, was developed and tested in the rats brain at 9.4 T. In particular, we focused on changing the truncation level of the HS1 pulse to generate different contributions of ${\text{T}}_{1\rho }$ and ${\text{T}}_{2\rho }$ relaxation pathways during the pulses. Our results demonstrate that the truncated HS1 pulses can be used *in vivo* with durations in sub-milliseconds time scale, which, in conjunction with zero-TE imaging readout, allow to probe fast decaying spins that are generally not accessible with conventional MRI relaxometry. Importantly, the truncated HS1 pulses operate with lower power settings than AFP pulses, a feature that greatly benefits translation of RETRO from preclinical work to humans. The presented theoretical treatment of relaxations due to dipole-dipole interactions and anisochronous exchange, along with the experimental *in vivo* demonstrations, provide a framework for exploiting the use of truncated HS1 pulses in living sample. Finally, while this work focused on altering the truncation level of HS1 pulses, frequency swept pulses can be designed with a variety of other modulation functions beyond those of the HS family; this opportunity is expected to greatly expand the flexibility of RETRO to alter the magnetization evolution and thus manipulate the MRI tissue contrast.

## Supplementary Material

MMC1

## Figures and Tables

**Table 1:** Correlation analyses between the relaxation rates and their differences, $\delta {\text{R}}_{1,2\rho }$, and optical density (OD) of myelin, iron and Nissl. The ODs used in the analysis were obtained from Hakkarainen et al. [22]. The analysis included regions: amygdala and piriform cortex (combined), corpus callosum, cingulum, dentate gyrus, fimbria, hippocampus, primary somatosensory cortex, optic tract, ventral posterior thalamic nuclei and posterior thalamic nucleus (combined). Pearson’s correlation coefficients and p-values (in parenthesis) are indicated in the table. Gray and white cells indicate results for the 4 metrics acquired with GRE readout, and the 7 metrics acquired with MB-SWIFT readout, respectively. Correlations that are significant after Bonferroni correction for multiple comparisons (n=4 or 7 comparisons) are highlighted in bold.

	Myelin	Nissl	Iron
${\text{R}}_{1\rho }\left(\text{t-HS}1-15{}^{\circ}\;6\;\text{ms}\right)$	r=−0.31	r=−0.03	r=0.19
	p=0.422	p=0.935	p=0.624
${\text{R}}_{1\rho }(\text{HS}1\;6\;\text{ms})$	r=−0.33	r=0.14	r=0.02
	p=0.389	p=0.715	p=0.968
${\text{R}}_{2\rho }\left(\text{t-HS}1-15{}^{\circ}\;6\;\text{ms}\right)$	r=−0.18	r=−0.18	r=0.18
	p=0.821	p=0.637	p=0.645
${\text{R}}_{2\rho }(\text{HS}1\;\text{6ms})$	r=−0.09	r=−0.30	r=0.35
	p=0.821	p=0.438	p=0.361
${\text{R}}_{1\rho }\left(\text{t-HS}1-15{}^{\circ}\;2\;\text{ms}\right)$	r=0.53	r=−0.50	r=0.33
	p=0.142	p=0.166	p=0.381
${\text{R}}_{1\rho }(\text{HS}1\;2\;\text{ms})$	r=0.53	r=−0.49	r=0.33
	p=0.144	p=0.183	p=0.388
${\text{R}}_{1\rho }\left(\text{t-HS}1-15{}^{\circ}\;1\;\text{ms}\right)$	r=0.63	r=−0.50	r=0.31
	p=0.067	p=0.171	p=0.423
${\text{R}}_{1\rho }\left(\text{t-HS}1-15{}^{\circ}\;0.7\;\text{ms}\right)$	r=0.64	r=−0.50	r=0.32
	p=0.063	p=0.167	p=0.394
${\text{R}}_{1\rho }\left(\text{t-HS}1-15{}^{\circ}\;2\;\text{ms}\right)-{\text{R}}_{1\rho }(\text{HS}1\;2\;\text{ms})$	r=0.4	r=−0.53	r=0.29
	p=0.286	p=0.14	p=0.456
$R_{1𝛒}(\text{t-HS}1-15{}^{\circ}\;1\;ms)-R_{1𝛒}(HS1\;2\;ms)$	**r=0.83**	r=−0.37	r=0.11
	**p=0.006**	p=0.329	p=0.788
${\text{R}}_{1\rho }\left(\text{t-HS}1-15{}^{\circ}\;0.7\;\text{ms}\right)-{\text{R}}_{1\rho }(\text{HS}1\;2\;\text{ms})$	r=0.75	r=−0.44	r=0.26
	p=0.019	p=0.231	p=0.507

## Data Availability

The data that support the findings of this study are openly available in Data Repository for the University of Minnesota (DRUM) at https://doi.org/10.13020/P7HS-HZ97.

## Appendix A. Supplementary data

Supplementary data to this article can be found online at https://doi.org/10.1016/j.jmr.2026.108115.

## Abbreviations:

RF: Radiofrequency
FS: Frequency swept
FRF: First rotating frame
RAFFn: Relaxations along a fictitious field in the rotating frame of rank n
AM: Amplitude modulation
FM: Frequency modulation
HS: Hyperbolic Secant
RETRO: RElaxation dependent on TRuncatiOn
AFP: Adiabatic full passage
WCR: Weak collision regime
ICR: Intermediate collision regime
SCR: Strong collision regime
HSn: Hyperbolic Secant pulses, n is stretching factor
AHP: Adiabatic half passage
SRF: Second rotating frame
R_1ρ_: Longitudinal rotating frame relaxation rate constant
R_2ρ_: Transverse rotating frame relaxation rate constant
