# Supplementary material for: Altering MRI rotating frame relaxations by changing the truncation level of Hyperbolic Secant pulse
Source: J Magn Reson. Author manuscript; Available in PMC 2026 Jul 18. (PMC13379954; doi:10.1016/j.jmr.2026.108115)
Supplement: MMC1 [file NIHMS2190696-supplement-MMC1.docx]

**Altering MRI rotating frame relaxations by changing the truncation level of Hyperbolic Secant pulse**

Sara Ponticorvo^1^, Lin Wu^1^, Andrej Lasica^2^, Hanne Laakso^3^, Dennis J. Sorce^4^, [Douglas L Rothman](https://pubmed.ncbi.nlm.nih.gov/?term=Rothman+DL&cauthor_id=34994222)^5^, Silvia Mangia^1^, Shalom Michaeli^1*^

^1^ Department of Radiology, Center for Magnetic Resonance Research (CMRR), University of Minnesota, Minneapolis, MN, USA.

^2^Department of Neurology, Charles University, First Faculty of Medicine and General University Hospital, Prague, Czech Republic.

^3^A. I. Virtanen Institute for Molecular Sciences, University of Eastern Finland, Kuopio, Finland

^4^ Independent Researcher, 6 Stonegate Court, Cockeysville, MD 21030, USA.

^5^ Department of Radiology and Biomedical Imaging, Magnetic Resonance Research Center (MRRC), Yale University, New Haven, CT, USA.

**
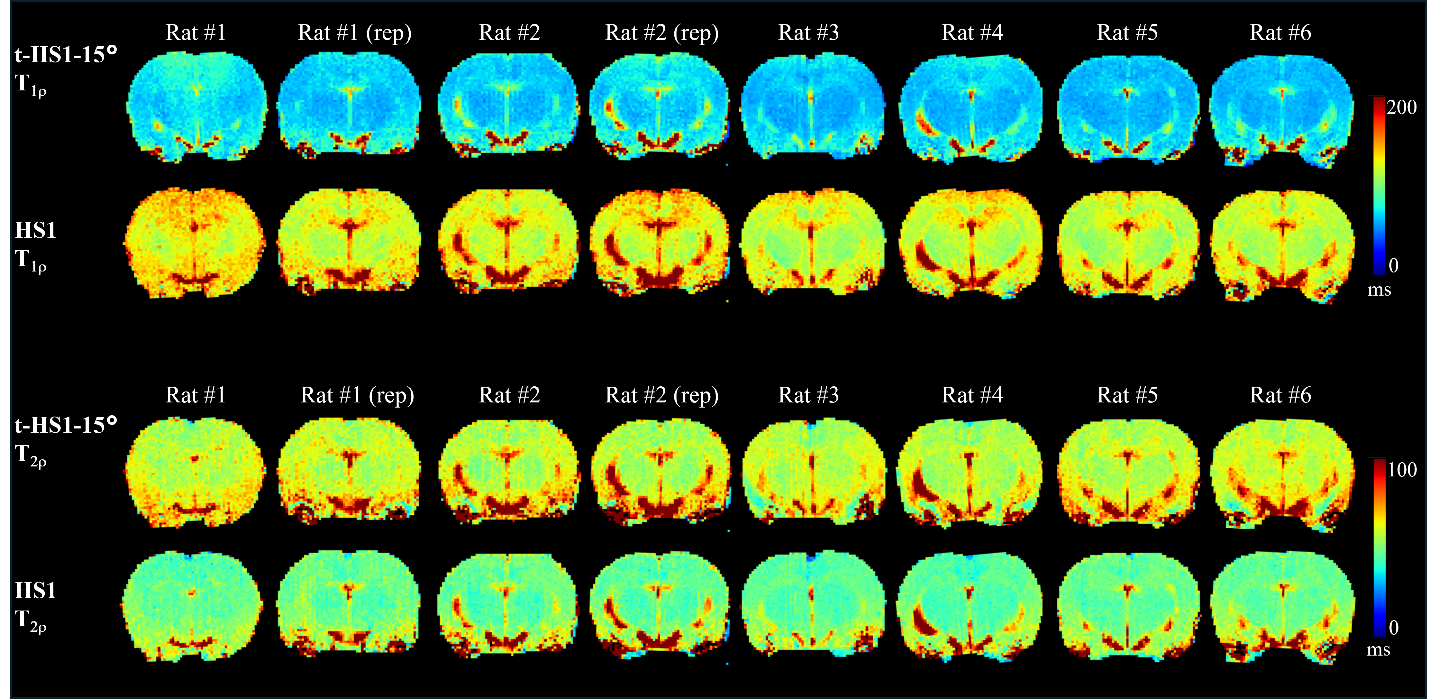
**

**Supplementary Figure 1.** Single-subject relaxation time constants maps using GRE readout. T_1ρ_ and T_2ρ_ maps were obtained with HS1 and t-HS1-15° pulses. Pulse duration T_p_=6 ms; ω_1_^max^/(2π)=2k Hz and 1.2 kHz for HS1 and t-HS1-15º pulses, respectively. A brain mask was applied for visualization purposes.

**
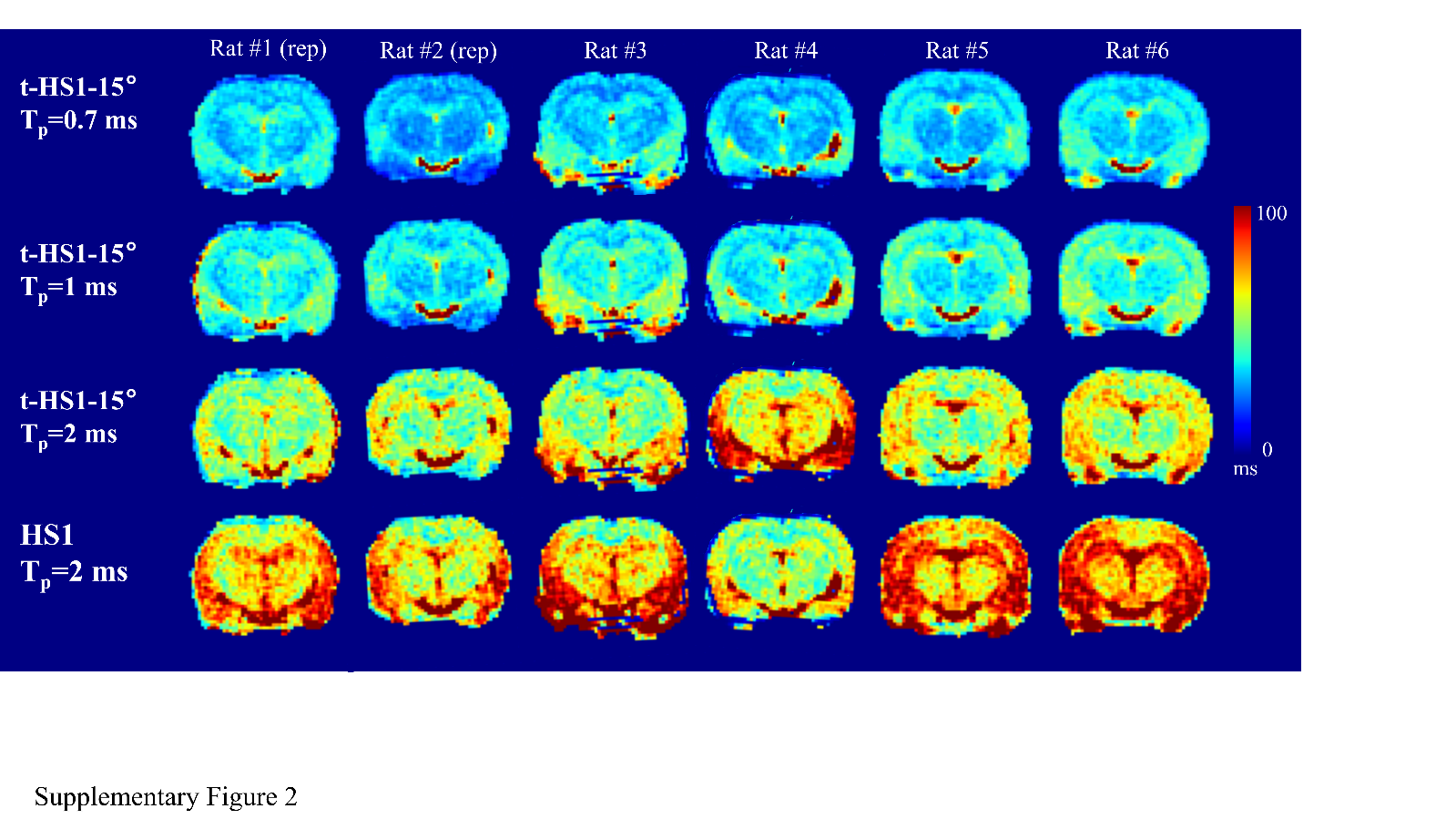
**

**Supplementary Figure 2.** Single-subject T_1ρ_ maps obtained with t-HS1-15° and HS1 pulses using zero-TE MB-SWIFT imaging read-out. The HS1 parameters were T_p_=2 ms and ω_1_^max^/(2π)=2.5 kHz; t-HS1-15º parameters were T_p_=2 ms and ω_1_^max^/(2π)=1.25 kHz, T_p_=1 ms and ω_1_^max^/(2π)=2.15 kHz, and T_p_=0.7 ms and ω_1_^max^/(2π)=3 kHz. For visualization purposes the maps have been spatially smoothed using FSL’s SUSAN noise reduction filter with a 1.16 mm kernel, and a brain mask was applied.
